# Factors affecting haemoglobin dynamics in African children with acute uncomplicated *Plasmodium falciparum* malaria treated with single low-dose primaquine or placebo

**DOI:** 10.1186/s12916-023-03105-0

**Published:** 2023-10-20

**Authors:** Marie A. Onyamboko, Peter Olupot-Olupot, Winifred Were, Cate Namayanja, Peter Onyas, Harriet Titin, Joy Baseke, Rita Muhindo, Daddy K. Kayembe, Pauline O. Ndjowo, Benjamin B. Basara, Charles B. Okalebo, Thomas N. Williams, Sophie Uyoga, Chiraporn Taya, Adeola Bamisaiye, Caterina Fanello, Kathryn Maitland, Nicholas P. J. Day, Walter R. J. Taylor, Mavuto Mukaka

**Affiliations:** 1 Kinshasa School of Public Health, University of Kinshasa, Avenue Tombalbaye 68-78, Kinshasa, Democratic Republic of Congo; 2 Busitema University, P.O. Box 1460, Mbale, Uganda; 3 Mbale Clinical Research Institute (MCRI), P.O. Box 1966, Mbale, Uganda; 4 KEMRI-Wellcome Trust Research Programme, Kilifi, Kenya; 5 Institute of Global Health Innovation, Department of Surgery and Cancer, Imperial College London, London, SW7 2AS UK; 6 Mahidol Oxford Tropical Medicine Research Unit (MORU), Faculty of Tropical Medicine, Mahidol University, 420/6 Rajvithi Road, Bangkok, 10400 Thailand; 7 Centre for Tropical Medicine and Global Health, Nuffield Department of Medicine, University of Oxford, Oxford, UK

**Keywords:** Primaquine, *Plasmodium falciparum*, Haemoglobin, Glucose-6-phosphate dehydrogenase deficiency, Anaemia, Children

## Abstract

**Background:**

Single low-dose primaquine (SLDPQ) effectively blocks the transmission of *Plasmodium falciparum* malaria, but anxiety remains regarding its haemolytic potential in patients with glucose-6-phopshate dehydrogenase (G6PD) deficiency. We, therefore, examined the independent effects of several factors on haemoglobin (Hb) dynamics in falciparum-infected children with a particular interest in SLDPQ and G6PD status.

**Methods:**

This randomised, double-blind, placebo-controlled, safety trial was conducted in Congolese and Ugandan children aged 6 months–11 years with acute uncomplicated *P. falciparum* and day (D) 0 Hbs ≥ 6 g/dL who were treated with age-dosed SLDPQ/placebo and weight-dosed artemether lumefantrine (AL) or dihydroartemisinin piperaquine (DHAPP). Genotyping defined G6PD (G6PD c.202T allele), haemoglobin S (HbS), and α-thalassaemia status.

Multivariable linear and logistic regression assessed factor independence for continuous Hb parameters and Hb recovery (D42 Hb > D0 Hb), respectively.

**Results:**

One thousand one hundred thirty-seven children, whose median age was 5 years, were randomised to receive: AL + SLDPQ (*n* = 286), AL + placebo (286), DHAPP + SLDPQ (283), and DHAPP + placebo (282). By G6PD status, 284 were G6PD deficient (239 hemizygous males, 45 homozygous females), 119 were heterozygous females, 418 and 299 were normal males and females, respectively, and 17 were of unknown status.

The mean D0 Hb was 10.6 (SD 1.6) g/dL and was lower in younger children with longer illnesses, lower mid-upper arm circumferences, splenomegaly, and α-thalassaemia trait, who were either G6PDd or heterozygous females. The initial fractional fall in Hb was greater in younger children with higher D0 Hbs and D0 parasitaemias and longer illnesses but less in sickle cell trait. Older G6PDd children with lower starting Hbs and greater factional falls were more likely to achieve Hb recovery, whilst lower D42 Hb concentrations were associated with younger G6PD normal children with lower fractional falls, sickle cell disease, α-thalassaemia silent carrier and trait, and late treatment failures. Ten blood transfusions were given in the first week (5 SLDPQ, 5 placebo).

**Conclusions:**

In these falciparum-infected African children, posttreatment Hb changes were unaffected by SLDPQ, and G6PDd patients had favourable posttreatment Hb changes and a higher probability of Hb recovery. These reassuring findings support SLDPQ deployment without G6PD screening in Africa.

**Trial registration:**

The trial is registered at ISRCTN 11594437.

**Supplementary Information:**

The online version contains supplementary material available at 10.1186/s12916-023-03105-0.

## Background

Since the 2012 WHO recommendation to use single low-dose primaquine (SLDPQ) as an elimination tool for multidrug, especially artemisinin, resistant *Plasmodium falciparum* (ARPf) [1], several studies have shown its transmission blocking efficacy and ability to reduce gametocyte carriage [2–6].

ARPf has been present in SE Asia for the past 15 years and has seen significant declines in efficacy of the partner drugs used in artemisinin-based combination treatments (ACTs) [7–11]. ARPf is now gaining a foothold in eastern Africa [12, 13] with concomitant evidence of a decline in the sensitivity of lumefantrine, a commonly used ACT [14, 15]. SLDPQ offers an opportunity to counter this threat, but concern remains that SLDPQ could increase the risk of life-threatening haemolysis and blood transfusions (primaquine’s most feared toxicity) in patients with glucose-6-phosphate dehydrogenase deficiency (G6PDd) with a consequential loss of credibility of this strategy by malaria control programmes (MCPs).

In sub-Saharan Africa, G6PD A-, caused by the c.202C → T mutation in the G6PD gene, is the most common variant with reported rates ranging from ~ 2–3% in The Gambia [16, 17]) to ~ 10% in Uganda [18], ~ 17% in Burkina Faso [19], and ~ 24% in Nigeria [20]. G6PDd is, thus, an important consideration for MCPs.

*Plasmodium falciparum* malaria itself causes haemolysis that may result in anaemia at presentation or following treatment with the mean Hb reaching its nadir concentration on day 2 [21, 22]. Accordingly, the highest risk of blood transfusion occurs early, within the first 4–7 days in falciparum-infected children < 5 years [23–25]. Reported blood transfusion rates were 1.5% (10/679) in ACT-treated children in the Democratic Republic of Congo [23], 1.4% (10/702) in Rwandan children treated with chlorproguanil-dapsone-artesunate (CDA) or amodiaquine-sulphadoxine-pyrimethamine [24], rising, in a Kenyan study, to 11% (13/119) in G6PDd children (1– < 15 years) vs. 0.5% (1/200) in heterozygous females treated with CDA or CD alone [25]. In western Thailand, the rate was 1% (55/5253) in children and adults given various treatments, principally, mefloquine alone or combined with artesunate [26].

There is growing evidence that SLDPQ is well tolerated across the age spectrum in patients, healthy individuals, and asymptomatic *P. falciparum* carriers with the African A- and the more severe Southeast Asian G6PD variants [27–33]. The mean difference in nadir Hb concentrations between SLDPQ treated G6PDd and G6PD normal malaria patients is ~ 1 g/dL [27, 28, 31], but at the higher primaquine doses used by Shekalaghe et al., 0.75 − 1.0 mg/kg, the mean difference was 2 g/dL between the G6PDd and G6PD normal children [34].

Our group recently published the largest study to date on the safety of a bespoke, age-dosed regimen of SLDPQ [35] in young G6PDd children with uncomplicated *P. falciparum*. SLDPQ was associated with the same blood transfusion rate as placebo (0.9%) and a very similar tolerability profile [36]. Extending our analysis of this study, we sought to determine the independent effects of several factors, including G6PD and sickle cell status, thalassaemia, and SLDPQ on the course of haemoglobin over time.

## Methods

### Study design, sites, and conduct

Briefly, in this randomised, placebo-controlled trial, children aged 6 months–11 years with uncomplicated *P. falciparum*, by blood slide or rapid diagnostic test, received either open artemether lumefantrine (AL) or dihydroartemisinin piperaquine (DHAPP), both dosed by weight, and aged-dosed SLDPQ/placebo: 1.25 mg (6 months– < 1 years), 2.5 mg (1–5 years), 5 mg (6–9 years), and 7.5 mg (10–14 years). A subgroup had intense blood sampling for pharmacokinetic (PK) analyses: day (D) 0 hour (H) 0 (baseline) and then at H1, H1.5, H2, H4, H8, H12, and H24 [37].

Genotyping was performed to identify the following: (i) the status of cytochrome (CYP) P450 2D6 [38, 39], (ii) G6PD A- by the *G6PD* c.202T allele [40], (iii) sickle cell trait (HbAS) and sickle cell disease (HbSS) [41], and (iv) α-thalassaemia caused by the common African-α^3.7^ deletion in *HBAA*, where children were classified as heterozygous (-α/αα), homozygous (-α/-α) or normal (αα/αα) [42].

Follow-up extended to D42, during which investigations included symptoms (to detect adverse events), Giemsa-stained blood films, venous blood (D0, 7, 14 and 28) for a complete blood count and routine biochemistry, and HemoCue measured Hb concentrations. The latter were used to define Hb changes over time: D0, 1, 2, 3, 7, 14, 21, 28, 35, and 42; in a subset of patients who were having intense monitoring of their parasite counts, additional samples were taken at D0 H1, H1.5, H2, H4, H6, H8, H12, H36, H60, H84, and H96.

The study sites, the Kinshasa Mahidol Oxford Research Unit (outside Kinshasa) and the Mbale Regional Referral Hospital (Mbale, SE Uganda), are in areas of hyperendemic, perennial malaria transmission of *P. falciparum* and both sites serve urban and rural populations; children < 5 years are the main risk group for malaria.

### Statistical analysis

The analysis population included all patients who received at least one dose of study drug and had at least one posttreatment Hb measurement. As per the WHO, anaemia of any degree was defined as a Hb < 11 g/dL in children < 5 years and < 11.5 mg/dL in those aged 5–11 years [43]; adapting the WHO definition, we defined moderate anaemia as a Hb < 8 g/dL for all ages.

Multivariable linear regression models assessed a range of clinically relevant variables that, collectively, have been assessed in previous studies [21–29, 44, 45]; these were age, sex, length of illness, mid upper arm circumference (MUAC), D0 Hb, D0 parasitaemia, splenomegaly, hepatomegaly, G6PD, HbS and α-thalassaemia genotype, ACT, SDLPQ/placebo, Hb fractional fall, and late treatment failure. We assessed their independent effects on the (i) D0 Hb concentration, (ii) initial decline in Hb concentration from D0 to the day of the nadir Hb in the first 14 days, (iii) nadir and D42 Hb concentrations, and (iv) increase in Hb from the nadir to D42 concentration, the total malaria attributable fall in Hb following treatment (MAFt, Additional file 1: Fig S1) [45], excluding variables where needed, e.g. ACT, SLDPQ/placebo, and late treatment failure were not used to assess the baseline Hb. In linear regression models, the slopes and the corresponding 95% confidence intervals for slopes are reported. Collinearity was assessed using the variance inflation factor (VIF) and variables with VIF > 5 would be excluded from the models. However, all variables had low VIFs (all < 3).

The factors associated with Hb recovery, defined as a D42 Hb > D0 Hb, were determined by logistic regression (age, sex, length of illness, MUAC, D0 Hb, D0 parasitaemia, splenomegaly, hepatomegaly, G6PD, HbS and α-thalassaemia genotype, ACT, SDLPQ/placebo, Hb fractional fall and late treatment failure). In these models, the odds ratios and the corresponding 95% confidence intervals for odds ratios are reported.

The times to Hb recovery by G6PD status were determined by the Kaplan–Meier survival approach, and potential explanatory variables for times to Hb recovery were explored by the Cox proportional hazards regression model. In these models, we used the scaled Schoenfeld residuals to test the proportional hazards assumption. Variables not meeting the proportional hazards assumptions were excluded from the analysis. The hazards ratios and the corresponding 95% confidence intervals for hazard ratios are reported. *P*-values have also been reported for all models considered.

A subgroup analysis (*n* = 258) is presented of patients with PK data to assess the effects of (i) the maximum primaquine concentration (*C*_max_), (ii) primaquine exposure, i.e. area under the drug concentration time curve from 0 to the last PK sample (AUC_0-t_), and (iii) mg/kg dose and CYP2D6 activity score, inferred from the CYP2D6 genotype [46]; C_max_ and AUC_0-t_ were determined by non-compartmental analysis. All statistical analyses were carried out using Stata v17 (Stata Corporation, Texas, USA) and were two sided. A *P*-value < 0.05 denoted statistical significance.

## Results

Between December 18, 2017, and October 7, 2019 (Mbale Regional Referral Hospital, Uganda), and July 17, 2017, and October 5, 2019 (Kinshasa Mahidol Oxford Research Unit, Kinshasa, DRC), 1137 children were recruited: 598 (52.6%) in Uganda and 539 (47.4%) in DRC. Baseline characteristics were comparable across all arms (Table 1). The median age and weight were 5 years and 17 kg, and the boy to girl ratio was 1.4:1. The total number of infants (aged 6 to < 12 months) was 55 (4.8%). Almost all children were of normal nutritional status (7 had moderate malnutrition and 1 severe acute malnutrition). G6PD status was established in 1120/1137 (98.5%) children; heterozygous α-thalassaemia was found in a little under half of children and homozygous α-thalassaemia in < 10%. HbAS was identified in 14.5%. There were two early treatment failures only in the AL and AL + placebo arms (2/542, 0.4%) and 101/542 (18.6%, AL arm) and 27/539 (5.1%, DHAPP arm) late treatment failures.

**Table 1:** Baseline characteristics of study participants

Variable	AL + PQ	AL + placebo	DHAPP + PQ	DHAPP + Placebo	Total
Number of participants	*N* = 286	*N* = 286	*N* = 283	*N* = 282	*N* = 1137
Age in years^a^	5.0 (3.0, 7.0)	5.0 (3.0, 7.0)	5.0 (3.0, 8.0)	5.0 (2.0, 8.0)	5.0 (3.0, 8.0)
Sex^b^					
Female	122 (42.7)	114 (39.9)	110 (38.9)	124 (44.0)	470 (41.3)
Male	164 (57.3)	172 (60.1)	173 (61.1)	158 (56.0)	667 (58.7)
Length of illness before treatment (days)^a^	2.0 (1.0, 3.0)	2.0 (2.0, 3.0)	2.0 (2.0, 3.0)	2.0 (2.0, 3.0)	2.0 (2.0, 3.0)
Fever (core temperature ≥ 38 °C)^b^	80 (28.0)	74 (25.9)	75 (26.5)	63 (22.4)	292 (25.7)
Mid-upper arm circumference (cm)^ac^	15.6 (14.5, 17.0) [11.0, 23.0]	15.6 (14.5, 17.0) [12.0, 28.0]	15.8 (14.5, 17.0) [12.0, 24.0]	15.5 (14.5, 16.8) [12.5, 21.0]	15.6 (14.5, 17.0) [11.0, 28.0]
Weight (Kg)^ac^	17.0 (12.8, 22.5) [5.9, 39.0]	17.0 (12.0, 22.0) [6.6, 41.5]	17.0 (12.9, 23.4) [6.6, 43.0]	16.5 (12.7, 22.0) [6.6, 35.7]	17.0 (12.7, 22.1) [5.9, 43.0]
Splenomegaly^b^	69 (24.1)	58 (20.3)	68 (24.0)	71 (25.2)	266 (23.4)
Hepatomegaly^b^	16 (5.6)	15 (5.2)	14 (4.9)	11 (3.9)	56 (4.9)
Microscopy^b^					
Positive slide result	249 (87.1)	251 (87.8)	239 (84.5)	246 (87.2)	985 (86.6)
Negative slide result	37 (12.9)	35 (12.2)	44 (15.5)	35 (12.5)	151 (13.3)
Mixed infection with					
*P. malariae* *n*	5	5	4	3	17
*P. ovale* *n*	-	-	-	1	1
Asexual parasitaemia (/μL)^d^	13,873 [7.2, 1,397,958.0]	12,345 [14.0, 1,100,764.0]	14,148 [12.0, 668,769.8]	18,848 [7.4, 2,172,060.0]	14,599 [7.2, 2,172,060.0]
Gametocytaemia (/μL) ^d^	52.6 [8.2, 1778.4]	36.9 [7.8, 2028.0]	49.9 [10.0, 2316.6]	44.0 [8.8, 2888.4]	45.5 [7.8, 2888.4]
Haemoglobin (g/dL)^e^	10.5 (1.7)	10.7 (1.5)	10.7 (1.6)	10.5 (1.6)	10.6 (1.6)^f^
Haematocrit (%) ^e^	31.0 (4.5)	31.5 (4.2)	31.7 (4.7)	30.9 (4.5)	31.3 (4.5)
G6PD status^b^					
Normal	176 (62.6)	186 (65.3)	174 (63.0)	181 (65.1)	717 (64.0)
Heterozygous female	32 (11.4)	32 (11.2)	24 (8.7)	31 (11.2)	119 (10.6)
Deficient males and females	73 (26.0)	67 (23.5)	78 (28.3)	66 (23.7)	284 (25.4)
Sickle cell status^b^					
Normal (HbAA)	248 (87.9)	243 (85.3)	234 (84.2)	231 (83.4)	956 (85.2)
Trait (HbAS)	33 (11.7)	42 (14.7)	42 (15.1)	46 (16.6)	163 (14.5)
Disease (HbSS)	1 (0.4)	0 (0.0)	2 (0.7)	0 (0.0)	3 (0.3)
Thalassaemia^b^					
Normal (αα/αα)	132 (46.8)	139 (48.9)	124 (45.6)	136 (48.9)	531 (47.6)
Heterozygous (-α/αα)	124 (44.0)	122 (43.0)	122 (44.9)	109 (39.2)	477 (42.7)
Homozygous (-α/-α)	26 (9.2)	23 (8.1)	26 (9.6)	33 (11.9)	108 (9.7)
Primaquine/placebo dose	0.21 (0.17, 0.25)	0.21 (0.18, 0.25)	0.20 (0.17, 0.25)	0.22 (0.18, 0.25)	0.21 (0.17, 0.25)
(mg/kg)^ac^	[0.11, 0.37]	[0.12, 0.35]	[0.07, 0.41]	[0.07, 0.38]	[0.07, 0.41]

^a^Median (IQR)

^b^*n* (%)

^c^Range [minimum, maximum]

^d^Geometric mean [range]

^e^Mean (SD)

^f^10.0 (1.5) g/dL (age < 5 years) vs. 11.1(1.5) g/dL (age 5–11 years), *p* < 0.001

**Fig. 1:**
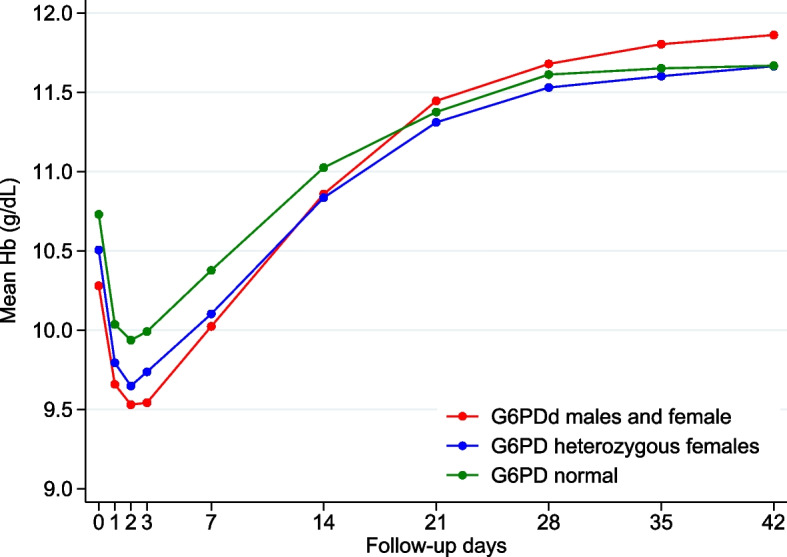
Changes in mean haemoglobin concentrations over time by G6PD status

**Fig. 2:**
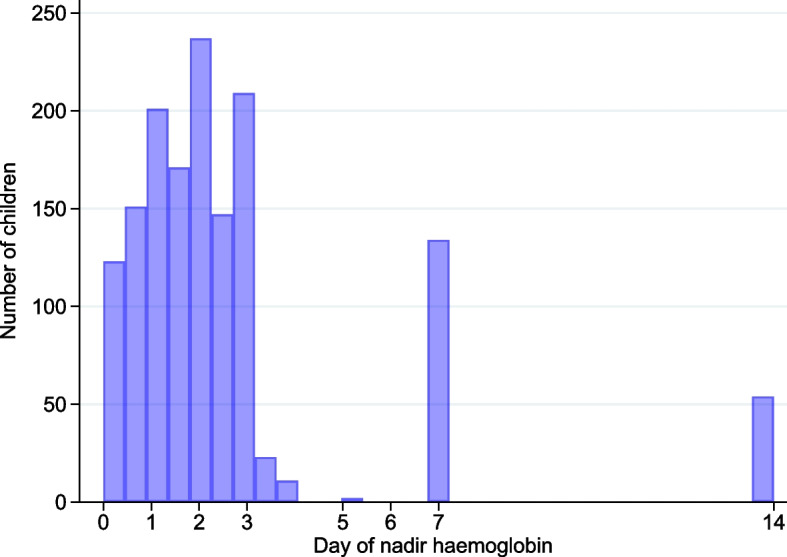
Histogram showing the days of the nadir haemoglobin concentrations up to day 14

The mean Hb fell initially to reach its mean nadir concentration on D2 and recovered by D14 in all children and when stratified by G6PD status (Fig. 1); the nadir Hb occurred as early as 6 h in 4.7% of children (54/1137, Fig. 2). A total of 3/1137 (0.3%) of children dropped their Hb to < 5 g/dL; all were transfused.

### Factors associated with baseline, nadir, and fractional fall in Hb

The baseline Hb was lower in younger children with a longer illness history, a lower MUAC, splenomegaly, who were homozygous α-thalassaemia, and either G6PDd or heterozygous females (Table 2). Although HbSS was associated with a significantly lower baseline Hb concentration, this result should be interpreted with caution because only 3 children were HbSS. The nadir Hb was higher with a higher baseline Hb, increasing age, and HbAS and lower in those with longer illness duration and higher baseline parasitaemias (Table 2).

**Table 2:** Factors associated with the haemoglobin concentrations at baseline, day of nadir, and on day 42 in 1137 patients and changes over time

**Variables**	**Multivariable analysis**	
	**Slope (95% CI)**	***P*-value**
Baseline haemoglobin (aR^2^ 0.2534)		
Age (years)	0.15 (0.11, 0.19)	< 0.001
Length of illness before treatment (days)	− 0.16 (− 0.23, − 0.09)	< 0.001
Mid-upper arm circumference (cm)	0.10 (0.04, 0.16)	0.001
Splenomegaly vs normal (reference)	− 1.07 (− 1.28, − 0.87)	< 0.001
*Sickle cell status*		
HbSS^a^ vs. HbAA (reference)	− 1.74 (− 3.33, − 0.16)	0.031
*Thalassaemia status*		
− α/ − α vs. αα/αα (reference)	− 0.67 (− 0.96, − 0.37)	< 0.001
*G6PD status*		
Heterozygous female vs. G6PD normal (reference)	− 0.30 (− 0.59, − 0.01)	0.043
Deficient vs. G6PD normal (reference)	− 0.34 (− 0.55, − 0.14)	0.001
**Nadir haemoglobin**^**b**^ **(aR**^**2**^**: 0.7299)**		
Age (years)	0.07 (0.05, 0.10)	< 0.001
Length of illness before treatment (days)	− 0.10 (− 0.15, − 0.06)	< 0.001
Baseline haemoglobin (g/dL)	0.72 (0.68, 0.75)	< 0.001
*Sickle cell status*		
HbAS v HbAA (reference)	0.25 (0.10, 0.40)	< 0.001
Log of baseline parasitaemia	− 0.10 (− 0.12, − 0.08)	< 0.001
**Fractional fall (%) in haemoglobin (aR**^**2**^**: 0.2149)**		
Age (years)	− 0.70 (− 0.93, − 0.47)	< 0.001
Length of illness before treatment (days)	0.98 (0.56, 1.41)	< 0.001
Baseline haemoglobin (g/dL)	1.39 (1.04, 1.73)	< 0.001
*Sickle cell status*		
HbAS v HbAA (reference)	− 2.28 (− 3.66, − 0.91)	0.001
Log of baseline parasitaemia	0.96 (0.78, 1.14)	< 0.001
**Total malaria attributable fraction (aR**^**2**^**: 0.5841)**		
Age (years)	0.11 (0.08, 0.14)	< 0.001
Length of illness before treatment (days)	0.57 (0.004, 0.11)	0.035
Fractional fall (%) in haemoglobin	− 0.81 (− 0.89, − 0.74)	< 0.001
Baseline haemoglobin (g/dL)	0.07 (0.10, 0.14)	0.024
*Sickle cell status*		
HbSS^a^ vs. HbAA (reference)	− 2.18 (− 3.97, − 0.39)	0.017
*G6PD status*		
Deficient vs. G6PD normal (reference)	0.18 (0.04, 0.32)	0.014
Log of baseline parasitaemia	0.03 (0.01, 0.06)	< 0.001
**Day 42 haemoglobin concentration (aR**^**2**^**: 0.3372)**		
Age (years)	0.13 (0.11, 0.15)	< 0.001
Haemoglobin fractional fall (%)	0.18 (0.11, 0.25)	< 0.001
*G6PD status*		
Deficient vs. G6PD normal (reference)	0.21 (0.07, 0.35)	0.003
*Sickle cell status*		
HbSS^a^ vs. HbAA (reference)	− 2.81 (− 4.12, − 1.51)	< 0.001
*Thalassaemia status*		
− α/αα vs. αα/αα (reference)	− 0.13 (− 0.25, − 0.01)	0.031
− α/ − α vs. αα/αα (reference)	− 0.37 (− 0.58, − 0.17)	< 0.001
Late treatment failure vs. ACPR (reference)	− 0.39 (− 0.56, − 0.21)	< 0.001
**Haemoglobin recovery by Day 42 (AIC: 752.92)**		
Age (years)	1.26 (1.17, 1.35)	< 0.001
Baseline haemoglobin (g/dL)	0.17 (0.13, 0.22)	< 0.001
Haemoglobin fractional fall (%)	1.76 (1.43, 2.17)	< 0.001
*G6PD status*		
Deficient vs. G6PD normal (reference)	1.68 (1.01, 2.78)	0.046

^a^*N* = 3 patients

^b^Mean Hb (SD) 8.6 (1.5) g/dL (age < 5 years) vs. 9.8 (1.3) g/dL (age 5–11 years), *p* < 0.001

The median fractional fall in Hb was 11.7% (IQR 6.8 − 17.5) with maximum falls of 37% (heterozygous females), 40.4% (G6PDd), and 57.9% (G6PD normal); these falls were greater in younger children with higher D0 Hbs and D0 parasitaemias and longer illnesses but less in HbAS (Table 2). The D0 Hb had the greatest influence on the fractional fall, but HbAS had a greater protective effect than increasing age.

### Factors associated with D42 Hb concentration and Hb recovery

A more robust MAFt response was seen in older G6PDd children with a longer illness duration and higher D0 Hb and D0 parasitaemia but was lower with a greater fractional fall in Hb and sickle cell disease (*n* = 3) vs. Hb AA. A higher D42 Hb concentration was seen in older G6PDd children with a greater fractional fall but was lower in patients with sickle cell disease (vs. Hb AA), heterozygous and homozygous α-thalassaemia, and those with late treatment failure. By D42, 807 of 1066 (75.7%) children recovered their Hb and were more likely to be older with G6PDd, lower D0 Hbs, and greater Hb fractional falls.

**Fig. 3:**
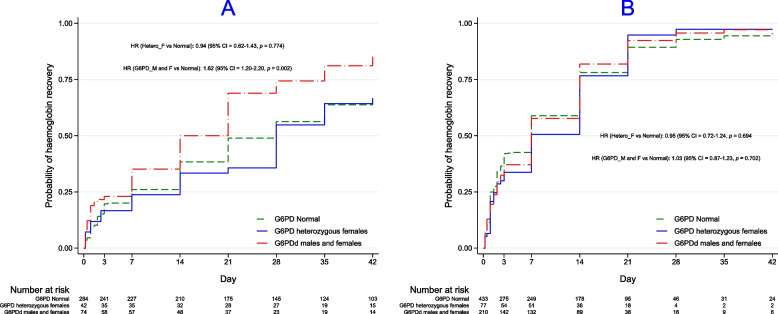
Times to haemoglobin recovery by G6PD status as a function of any degree of baseline anaemia (**A** = not anaemic, **B** = anaemic)

The time to Hb recovery ranged from 6 h − 42 days for a median of 14 (IQR 2–21) days and 7 (2 − 21) days in the G6PDd group. However, there was an interaction between baseline Hb and G6PD status on the time to haemoglobin recovery, suggesting a differential time to recovery in the G6PD-deficient group that depended on Hb concentration (Additional file 2: Tab S1). Therefore, we stratified these analyses by the presence/absence of anaemia of any degree to understand better the effect of the observed significant interaction. The time to Hb recovery was significantly faster in the G6PDd children without anaemia, but there was no difference by G6PD status in the anaemic group (Fig. 3). This was confirmed in the Cox proportional hazards regression model, which also showed that there were no other explanatory variables (Additional file 2: Tab S1). MUAC, fractional fall, splenomegaly, and log baseline parasitaemia violated the proportional hazards assumption in the univariate analyses and were excluded from the multivariable analysis (all were significant in the univariate analysis).

Being on SLDPQ was not an independent explanatory factor for the time to and probability of Hb recovery by D42 and the D42 Hb concentration.

### Effect of primaquine pharmacokinetics and activity score

For all the postbaseline parameters of haemoglobin dynamics, there was no effect of primaquine when modelled either as a categorical factor, i.e. ACT + SLDPQ vs. ACT alone in all patients (Table 2), or in the PK subgroup (Additional file 3: Tab S2) when expressed as the mg/kg dose with CYP2D6 activity score (Additional file 3: Tab S3-8), AUC_0-t_ (Additional file 3: Tab S9-14), or as the C_max_ (Additional file 3: Tab S15-20).

### Hb dynamics in children with normal Hbs or moderate anaemia at baseline

**Fig. 4:**
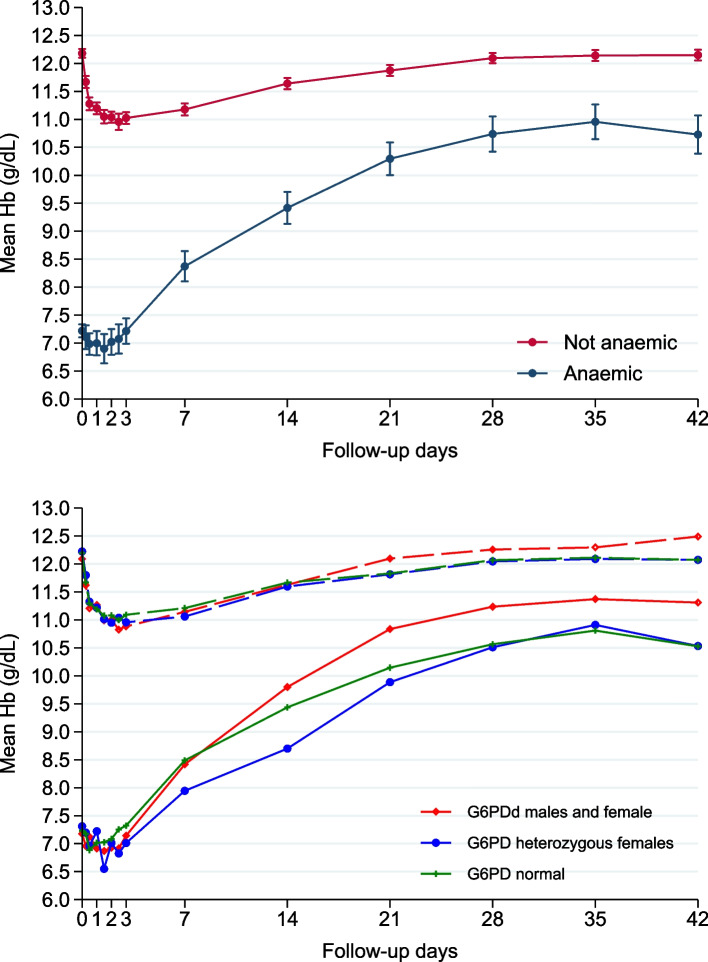
Mean haemoglobin changes over time with 95% confidence intervals for all children with normal baseline haemoglobin concentrations or moderate anaemia and shown by G6PD status. There were 407 patients without baseline anaemia, defined as a haemoglobin ≥ 11 g/dL for children < 5 years and ≥ 11.5 mg/dL for 5–11 years: G6PD normal = 284 (70%), G6PD heterozygous females = 42 (10%), G6PDd males and females = 74 (18%), and 7 with missing G6PD data. Patients with moderate anaemia (haemoglobin < 8 mg/dL) numbered 76: G6PD normal = 44 (58%), G6PD heterozygous females = 9 (12%), G6PDd males and females = 22 (29%), and 1 with missing G6PD data

There were 407 patients without baseline anaemia and 76 with moderate anaemia (Hb < 8 g/dL). G6PD status is detailed in Fig. 4. There was a very small, initial decline in the mean Hb and more rapid recovery and robust mean MAFt in the moderately anaemic children, with a greater response in the G6PDd group.

### Blood transfusions

There were 10 blood transfusions (0.9%) in the first week, equally distributed between SLDPQ and placebo, and one transfusion on D31 in a patient with recurrent parasitaemia (Table 3). The 3 children with Hbs < 5 g/dL had fractional falls of 23 to 58%. None of the HbAS or HbSS children were transfused.

**Table 3:** Patients who were transfused. None had sickle cell trait or sickle cell disease

Day of blood transfusion	Age	Sex	Enlarged spleen	D0 parasitaemia (/µL)	G6PD status	Thalassaemia status^a^	SLDPQ/placebo	ACT	D0 Hb (g/dL)	Hb when transfused day (g/dL)	Fractional fall	Develop severe malaria	Parasitaemia when transfused/µL)
D0	3	Male	N	0^b^	DEF	αα/αα	SLDPQ	DHAPP	6.5	4.8	− 0.26	Y—severe anaemia	0
D0	3	Male	Y	0^b^	NOR	-α/αα	Placebo	AL	6.4	4.9	− 0.23	Y—severe anaemia	0
D1	3	Male	N	28,786	NOR	-α/αα	SLDPQ	DHAPP	11.4	4.8	− 0.58	Y—prostration and severe anaemia	98
D1	1	Male	N	660,970	DEF	αα/αα	Placebo	AL	8.3	5.9	− 0.29	Y—unrousable coma	586,678
D2	1	Male	N	250,536	NOR	αα/αα	SLDPQ	DHAPP	8	4.6	− 0.43	N	0
D2	2	Female	Y	579,443	NOR	-α/αα	SLDPQ	AL	6.8	4.8	− 0.29	N	203
D2	6	Female	Y	211,430	NOR	αα/αα	Placebo	AL	9.4	4.9	− 0.48	N	0
D4	9	Female	Y	497,818	DEF	αα/αα	Placebo	AL	6.5	6.5	0.00	N	0
D4	4	Male	N	260	DEF	αα/αα	SLDPQ	AL	6.5	5.3 (1 day before)	− 0.18	Y—prostration and severe anaemia	0 (1 day before)
D5	4	Male	Y	5860	NOR	-α/αα	Placebo	DHAPP	9.8	6.7	− 0.32	N	0
D31	9	Female	N	219,637	NOR	-α/αα	SLDPQ	DHAPP	13.3	9.6 (4 days before)	− 0.28	Y—prostration, haemoglobinuria, and severe anaemia	1908 (4 days before)

^a^αα/αα normal haemoglobin type, -α/αα heterozygous alpha thalassaemia (silent carrier), -α/-α homozygous alpha thalassaemia (thalassaemia trait)

^b^Malaria slide negative but rapid test positive

## Discussion

MCPs with residual anxiety regarding the safety of SLDPQ should be reassured by the substantial evidence from our earlier randomised, placebo-controlled trial, which showed that the toxicity of age-dosed SLDPQ was similar to that of placebo and significantly reduced gametocyte carriage [36]. Moreover, despite the resulting higher PQ exposure [37] compared to SLDPQ in asymptomatic *P. falciparum* carriers [47], SLDPQ was well tolerated and, as we show here, had no deleterious effects on any of the posttreatment Hb parameters.

In this analysis, we detail the factors influencing Hb dynamics to guide clinicians and MCPs on identifying particular patients who may be at increased risk of developing clinically significant and, possibly, life-threatening anaemia and those who may not recover their Hb by 6 weeks.

At baseline, children with a lower Hb concentration were younger, had longer illness durations, lower MUACs, an increased prevalence of splenomegaly, and were more frequently affected by either homozygous α-thalassaemia, HbSS, or G6PDd (either as heterozygous females or hemizygous males/homozygous females). Consistent with previous clinical descriptions [48], baseline Hb appeared lowest among children with HbSS, although the small numbers affected precluded meaningful further analysis. Given the high frequency of HbSS of ~ 1–2% at birth [49, 50], the low frequency of HbSS among children recruited to this trial (3/1137; 0.26%) is striking, particularly given our recent observation that ~ 30% of children recruited to a recent trial of severe anaemia (one site was Mbale) had HbSS [51]. This low HbSS rate can be explained by several factors. Children with Hbs < 6 g/dL were excluded; HbSS-affected children [51, 52], like those with HbAS [53], are innately resistant to *P. falciparum* infections but suffer high rates (> 50%) of early-life mortality [54].

Some of our findings, notably age, length of illness, and splenomegaly, were also significant factors for baseline Hb or anaemia in African patients, especially in children < 5 years [21], in children and adults in Thailand [26], and in adults in Indonesia [45]. We found no association between baseline Hb and baseline parasite count in contrast to WWARN who reported an inverse relationship until a parasitaemia of 10,000/μL, followed by a positive relationship [22], whilst Zwang et al. reported a weak positive relationship between baseline Hb and baseline parasitaemia in children < 5 years (mean 0.08 g/dL/year) but an inverse relationship in patients aged ≥ 5 years [21].

The majority of patients with uncomplicated falciparum infections experience an initial fall in Hb and reach their nadir concentration on day 2 [21, 22] and the first posttreatment week is the time of highest risk for a blood transfusion [23–25, 36, 55]. Therefore, the fractional decline and the nadir Hbs are important parameters to consider in conjunction with clinical signs of severity before deciding the need for a blood transfusion. Previous work has also identified hyperparasitaemia (≥ 150,000/μL) [23] and giving CDA or CD to G6PDd males and heterozygous females [24, 25] as risk factors for a blood transfusion.

We found that a lower nadir Hb was associated with a lower baseline Hb in younger children with longer illnesses and higher baseline parasite counts; these factors were also associated with greater fractional falls in Hb except that a higher baseline Hb was associated with a greater fractional fall. Importantly, SLDPQ, G6PDd, and α-thalassaemia genotypes were not explanatory factors for both parameters. HbAS was associated with a slightly higher nadir Hb and lower fractional fall compared to those with normal Hbs that may be related to greater pretreatment haemolysis and, therefore, fewer red cells are available to haemolyse after treatment is given. Our findings concur with previous work showing that younger age, higher baseline Hbs and parasitaemias, and length of illness are significantly associated with a greater mean fractional fall in Hb [21, 22, 26, 27, 45], although Zwang et al. found age was not a factor [21].

Few studies have examined the effects of inherited blood disorders on malaria dynamics. Genotypically defined G6PDd and CYP2D6 genotypes were not factors in the initial absolute fall in falciparum-infected Tanzanian patients (median age 6.4 years), who were treated with SLDPQ [56], and α-thalassaemia genotype was not a factor in the D7 fractional fall in Hb in Tanzanian children (median age 4 years) [34]. In a small study from Cambodia, there was no association between the fractional fall in Hb and G6PD Viangchan and HbE status [27].

Posttreatment Hb recovery occurs as the malaria-induced bone marrow dyserythropoiesis and suppression are reversed and the haemolysis of parasitised and non-parasitised red cells ceases [57]. The return of bone marrow function is reflected by the MAFt, the time to Hb recovery, and an increasing reticulocyte count; the latter peaked on D7 in our patients and was independent of G6PD status [36].

Although we observed a rapid median time to Hb recovery (14 days), ~ 25% of children did not recover their Hb. A higher baseline Hb resulted in a longer time to Hb recovery (Fig. 4), consistent with Zwang et al. [21], a lower probability of achieving Hb recovery, and a lower MAFt (Fig. 4); the latter was also reported in a small study of falciparum-infected Papuan adults [45]. By contrast, our multivariable analysis found a higher D0 Hb was associated with a higher, albeit very small, mean increase in MAFt (Table 2), and this discrepancy might be a chance finding. Increasing age, a higher initial fractional fall and being G6PDd were associated with achieving a higher D42 Hb concentration and, in addition to a lower D0 Hb, a greater probability of Hb recovery by D42. G6PDd children had a more rapid time to Hb recovery, only if they had normal baseline Hbs, ~ 70% increased likelihood of D42 Hb recovery compared to G6PD normal children, and achieved a modest increase in mean D42 Hb concentration of 0.2 g/dL.

Despite the apparent disadvantage of a lower baseline Hb, the D42 Hb concentration was not affected, in contrast to a small study of adults from Papua [45]. However, a lower D42 Hb concentration was associated with either α-thalassaemia genotype (potentially reflecting normal physiology), sickle cell disease, or failing treatment; whilst the latter has been previously reported in two studies [26, 45], it was not a factor in Zwang et al.’s meta-analysis of posttreatment anaemia in patients of all ages [21].

Our study had several limitations. The number of children with moderate anaemia was quite low (~ 7%); there were only three children with HbSS (cautioning interpretation of significant findings), and the majority of children had good nutritional status. As a result, we cannot generalise our findings to children in the community with Hb concentrations of 4–6 g/dL and uncomplicated disease, those with HbSS, or those with moderate or severe acute malnutrition. We dosed SLDPQ by age, which is typically associated with broader mg/kg doses compared to weight-based dosing [35], so our findings may differ from studies based on weight-based regimens. Finally, we conducted many analyses, meaning that some significant findings (e.g. the association between the baseline Hb and the MAFt) might have reflected chance.

## Conclusions

To conclude, our large study has shown clearly that SLDPQ did not affect any of the posttreatment parameters of Hb dynamics and has provided additional reassuring evidence of its tolerability. Moreover, G6PDd was only associated with one negative finding—a lower mean baseline Hb concentration. Thereafter, it had a positive posttreatment effect on achieving Hb recovery and a higher D42 Hb but, crucially, did not affect the nadir Hb or the initial fall in Hb. Moreover, children with a Hb < 8 g/dL had a robust posttreatment Hb recovery, allaying anxieties that a lower baseline Hb is necessarily harmful.

Our findings strongly support the notion that using SDLPQ in G6PDd patients is safe and does not increase the risk of subsequent blood transfusion. Indeed, equal numbers of G6PDd and G6PD normal patients were transfused and our overall transfusion rate was just under 1% [36]. SLDPQ should be deployed more widely in Africa as part of a global strategy to eliminate ARPf.

### Supplementary Information

**Additional file 1: Figure S1.** Changes in the mean haemoglobin concentrations showing the malaria attributable fractions in all treated children, irrespective of G6PD status and treatment.

**Additional file 2: Table S1.** Factors associated with time to haemoglobin recovery by Cox Proportional Hazards model.

**Additional file 3: Table S2.** Baseline characteristics of patients in the PK substudy. **Tables S3-20.** Risk factors, including PK parameters, for haematological outcomes in the PK patient subset.

## Data Availability

We have provided much detailed analysis in this paper. Nevertheless, deidentified individual participant data and relevant supplementary data and documents (e.g., data dictionary, protocol, and participant information sheet) will be available to applicants who provide a sound proposal to the Mahidol Oxford Tropical Medicine Research Unit Data Access Committee (datasharing@tropmedres.ac). A data access agreement will be put in place before sharing.

## Abbreviations

A: Sub-Saharan Africa
ACT: Artemisinin-based combination therapy
AL: Artemether-lumefantrine
ARPf: Artemisinin resistant *Plasmodium falciparum*
AS: Artesunate
AUC_0-t_: Area under the drug concentration time curve from 0 to the last PK sample
CD: Chlorproguanil dapsone
CDA: Chlorproguanil-dapsone-artesunate
CI: Confidence interval
C_max_: Maximum concentration
D: Day
DHAPP: Dihydroartemisinin-piperaquine
IQR: Interquartile range
G6PDd: Glucose-6-phosphate dehydrogenase deficiency
Hb: Haemoglobin
HbAA: Normal haemoglobin
HbAS: Sickle cell trait
HbSS: Sickle cell disease
MAFt: Total malaria attributable fall in Hb following treatment
MUAC: Mid upper arm circumference
PK: Pharmacokinetics
PQ: Primaquine
SLDPQ: Single low-dose primaquine
WHO: World Health Organization
WWARN: Worldwide Antimalarial Resistance Network
