# Supplementary material for: Factors affecting haemoglobin dynamics in African children with acute uncomplicated Plasmodium falciparum malaria treated with single low-dose primaquine or placebo
Source: BMC Med. 2023 Oct 20;21:397. doi: 10.1186/s12916-023-03105-0 (PMC10588240; doi:10.1186/s12916-023-03105-0)

Figure S1. Changes in the mean haemoglobin concentrations showing the malaria attributable fractions in all treated children, irrespective of G6PD status and treatment.

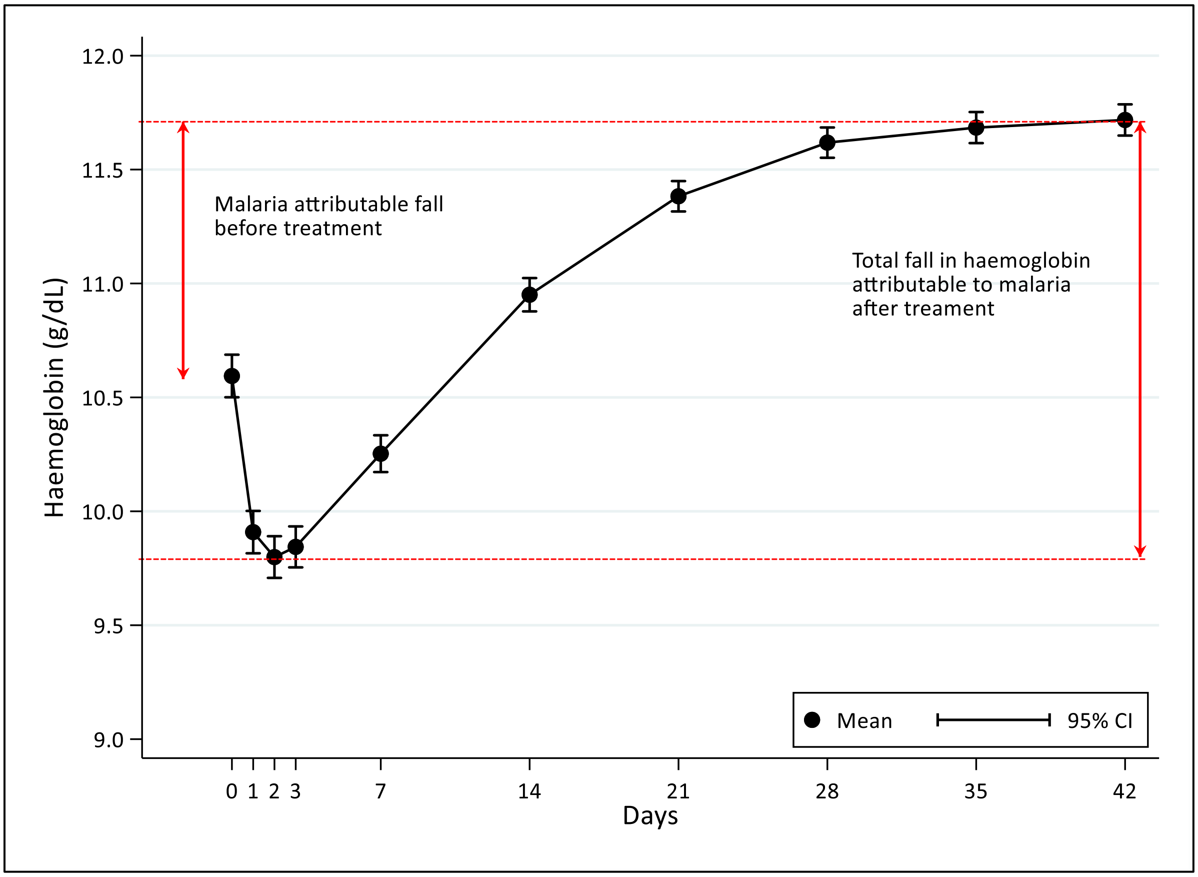

Supplement: Supplementary file 1 — Additional file 1: Figure S1. Changes in the mean haemoglobin concentrations showing the malaria attributable fractions in all treated children, irrespective of G6PD status and treatment. [file 12916_2023_3105_MOESM1_ESM.pdf]
