# Supplementary material for: Factors affecting haemoglobin dynamics in African children with acute uncomplicated Plasmodium falciparum malaria treated with single low-dose primaquine or placebo
Source: BMC Med. 2023 Oct 20;21:397. doi: 10.1186/s12916-023-03105-0 (PMC10588240; doi:10.1186/s12916-023-03105-0)
Supplement: Supplementary file 2 — Additional file 2: Table S1. Factors associated with time to haemoglobin recovery by Cox Proportional Hazards model. [file 12916_2023_3105_MOESM2_ESM.docx]

Additional file 2.

# Table S1. Factors associated with time to haemoglobin recovery by Cox Proportional Hazards model as a function of anaemia at baseline (N=1,137).

| **Variables** | **Univariate analysis** | | **Multivariable analysis** | |
| --- | --- | --- | --- | --- |
|  | **HR (95% CI)** | **p-value** | **Adjusted HR (95% CI)** | **p-value** |
| **Non anaemic group (n=407)** |  |  |  |  |
|  |  |  |  |  |
| Age (years) | 0.99 (0.96, 1.03) | 0.772 | 0.99 (0.95, 1.03) | 0.537 |
| Sex  Male (reference)  Female | 0.91 (0.72, 1.15) | 0.419 | 1.03 (0.79, 1.35) | 0.824 |
| Length of illness before treatment (days) | 0.97 (0.89, 1.07) | 0.575 | NA | NA |
| Sickle cell status  Normal (HbAA) (reference) |  |  |  |  |
| Trait (HbAS) | 1.14 (0.83, 1.55) | 0.426 | NA | NA |
| Disease (HbSS)* | NA | NA | NA | NA |
| Thalassemia status  Normal (αα/αα) (reference) |  |  |  |  |
| Heterozygous (-α/αα) | 0.81 (0.63, 1.03) | 0.092 | NA | NA |
| Homozygous (-α/-α) | 1.04 (0.63, 1.71) | 0.891 | NA | NA |
| Treatment failure  ACPR (reference) | 1.26 (0.87, 1.82) | 0.218 | NA | NA |
| ACT+ SLDPQ  ACT+ placebo (reference) | 1.00 (0.79, 1.26) | 0.992 | 1.01 (0.80, 1.28) | 0.922 |
| G6PD  Normal (reference) |  |  |  |  |
| Heterozygous females | 0.96 (0.65, 1.43) | 0.842 | 0.94 (0.62, 1.43) | 0.774 |
| **Deficient males and females** | **1.59 (1.20, 2.12)** | **0.001** | **1.62 (1.20, 2.20)** | **0.002** |
|  |  |  |  |  |
| **Anaemic group (n=730)** |  |  |  |  |
|  |  |  |  |  |
| Age (years) | 1.02 (0.99, 1.04) | 0.220 | 1.01 (0.99, 1.04) | 0.268 |
| Sex  Male (reference)  Female | 0.99 (0.85, 1.15) | 0.902 | 1.02 (0.85, 1.21) | 0.843 |
| Length of illness before treatment (days) | 0.99 (0.93, 1.05) | 0.866 | NA | NA |
| Sickle cell status  Normal (HbAA) (reference) |  |  |  |  |
| Trait (HbAS) | 1.04 (0.84, 1.29) | 0.726 | NA | NA |
| Disease (HbSS)* | 0.42 (0.10, 1.68) | 0.220 | NA | NA |
| Thalassemia status  Normal (αα/αα) (reference) |  |  |  |  |
| Heterozygous (-α/αα) | 0.98 (0.83, 1.15) | 0.790 | NA | NA |
| Homozygous (-α/-α) | 1.03 (0.81, 1.31) | 0.803 | NA | NA |
| Treatment failure  ACPR (reference) | 1.18 (0.94, 1.47) | 0.151 | NA | NA |
| ACT+ SLDPQ  ACT+ placebo (reference) | 1.00 (0.86, 1.15) | 0.954 | 0.98 (0.84, 1.13) | 0.752 |
| G6PD  Normal (reference) |  |  |  |  |
| Heterozygous females | 0.82 (0.40, 1.69) | 0.591 | 0.95 (0.72, 1.24) | 0.694 |
| Deficient males and females | 0.93 (0.55, 1.60) | 0.804 | 1.03 (0.87, 1.23) | 0.702 |
|  |  |  |  |  |
| **Interaction test** |  |  |  |  |
|  |  |  |  |  |
| G6PD Status and Baseline haemoglobin interaction: |  |  |  |  |
| Heterozygous females and Baseline   haemoglobin interaction |  |  | 1.04 (0.92, 1.18) | 0.486 |
| **Deficient males and females and   Baseline haemoglobin interaction** |  |  | **1.15 (1.05, 1.26)** | **0.002** |

* N=3 patients

Table S1 Akaike Information Criterion (AIC) for not anaemic model: 3,101.55

Table S1 Akaike Information Criterion (AIC) for anaemic model: 539.27

Table S1 Akaike Information Criterion (AIC) for interaction model: 12,271.57
