# Supplementary material for: Factors affecting haemoglobin dynamics in African children with acute uncomplicated Plasmodium falciparum malaria treated with single low-dose primaquine or placebo
Source: BMC Med. 2023 Oct 20;21:397. doi: 10.1186/s12916-023-03105-0 (PMC10588240; doi:10.1186/s12916-023-03105-0)
Supplement: Supplementary file 3 — Additional file 3: Table S2. Baseline characteristics of patients in the PK substudy. Tables S3-20. Risk factors, including PK parameters, for haematological outcomes in the PK patient subset. [file 12916_2023_3105_MOESM3_ESM.docx]

Additional file 3.

[Table S2. Baseline characteristics of participants in the pharmacokinetic analysis (N=258). 2](#_Toc147092814)

[Table S3. Factors associated with nadir haemoglobin, including mg/kg dose of primaquine and CYP2D6 activity score (N=258). 4](#_Toc147092815)

[Table S4. Factors associated with fractional fall (%) in haemoglobin, including mg/kg dose of primaquine and CYP2D6 activity score (N=258). 5](#_Toc147092816)

[Table S5. Factors associated with haemoglobin concentration at day 42 including mg/kg dose of primaquine and CYP2D6 activity score (N=258). 6](#_Toc147092817)

[Table S6. Factors associated with haemoglobin recovery on Day 42, including mg/kg dose of primaquine and CYP2D6 activity score (N=258). 7](#_Toc147092818)

[Table S7. Factors associated with malaria attributable fraction, including mg/kg dose of primaquine and CYP2D6 activity score (N=258). 8](#_Toc147092819)

[Table S8. Factors associated with time to haemoglobin recovery, including mg/kg dose of primaquine and CYP2D6 activity score (N=258). 9](#_Toc147092820)

[Table S9. Factors associated with nadir haemoglobin, including primaquine AUC_0-t_ (N=258). 10](#_Toc147092821)

[Table S10. Factors associated with fractional fall (%) in haemoglobin, including primaquine AUC_0-t_ (N=258). 11](#_Toc147092822)

[Table S11. Factors associated with haemoglobin concentration at day 42, including primaquine AUC_0-t_ (N=258). 12](#_Toc147092823)

[Table S12. Factors associated with haemoglobin recovery at day 42, including primaquine AUC_0-t_ (N=258). 13](#_Toc147092824)

[Table S13. Factors associated with malaria attributable fraction, including primaquine AUC_0-t_ (N=258). 14](#_Toc147092825)

[Table S14. Factors associated with time to haemoglobin recovery, including primaquine AUC_0-t_ (N=258). 15](#_Toc147092826)

[Table S15. Factors associated with nadir haemoglobin, including primaquine *C*_max_ (N=258). 16](#_Toc147092827)

[Table S16. Factors associated with fractional Fall (%) in haemoglobin, including primaquine *C*_max_ (N=258). 17](#_Toc147092828)

[Table S17. Factors associated with haemoglobin concentration at day 42, including primaquine *C*_max_ (N=258). 18](#_Toc147092829)

[Table S18. Factors associated with haemoglobin recovery at day 42, including primaquine *C*_max_ (N=258). 19](#_Toc147092830)

[Table S19. Factors associated with malaria attributable fraction, including primaquine *C*_max_ (N=258). 20](#_Toc147092831)

[Table S20. Factors associated with time to haemoglobin recovery, including primaquine *C*_max_ (N=258). 21](#_Toc147092832)

[Table S21 (A) Mean /Median /IQR /Range for haemoglobin parameters (Total N=1,137). 22](#_Toc147092833)

# Table S2. Baseline characteristics of participants in the pharmacokinetic analysis (N=258).

| **Variable** | **AL + PQ** | **DHAPP + PQ** | **Total** |
| --- | --- | --- | --- |
| Number of participants | N=128 | N=130 | N=258 |
| Country†  Uganda  DRC | 78 (60.9)  50 (39.1) | 78 (60.0)  52 (40.0) | 156 (60.5)  102 (39.5) |
| Age in years* | 5.0 (3.0, 7.0) | 5.0 (3.0, 8.0) | 5.0 (3.0, 7.0) |
| Sex†  Female | 74 (57.8) | 83 (63.8) | 157 (60.9) |
| Male | 54 (42.2) | 47 (36.2) | 101 (39.1) |
| Length of illness before treatment (days)* | 2.0 (2.0, 3.0) | 2.0 (2.0, 3.0) | 2.0 (2.0, 3.0) |
| Fever (core temperature ≥38°C)† | 31 (24.0) | 36 (28.0) | 67 (26.0) |
| Mid-upper arm circumference (cm)*^§^ | 15.6 (14.5, 17.0)  [11.0, 22.1] | 15.8 (14.5, 17.0)  [12.0, 21.5] | 15.6 (14.5, 17.0)  [11.0, 22.1] |
| Weight (Kg)*^§^ | 17.2 (13.8, 22.5) | 17.8 (13.0, 23.6) | 17.3 (13.3, 23.0) |
|  | [7.0, 39.0] | [6.6, 42.0] | [6.6, 42.0] |
| Splenomegaly†  Present  Absent | 100 (78.1)  28 (21.9) | 103 (79.2)  27 (20.8) | 203 (78.7)  55 (21.3) |
| Hepatomegaly†  Present  Absent | 123 (96.1)  5 (3.9) | 126 (96.9)  4 (3.1) | 249 (96.5)  9 (3.5) |
| Malaria test†  Positive RDT | 128 (100.0) | 130 (100.0) | 258 (100.0) |
| Microscopy†  Positive slide result  Negative slide result | 106 (83.0)  22 (17.0) | 102 (78.0)  28 (22.0) | 208 (81.0)  50 (19.0) |
| Mixed infection with |  |  |  |
| *P. malariae* n | 1 | 2 | 3 |
| *P. ovale*  n | - | - | - |
| Asexual parasitaemia (/µL)^#^ | 10,263.9  [8.8, 367,380.0] | 19,802.5  [19.6, 668,769.8] | 14,256.6  [8.8, 668,769.8] |
| Gametocytaemia (/µL)^#^ | 44.6  [8.2, 1,050.6] | 50.1  [11.2, 2,244.8] | 46.9  [8.2, 2,244.8] |
| Haemoglobin (g/dL)^¶^ | 10.4 (1.7) | 10.6 (1.6) | 10.5 (1.7) |
| Haematocrit (%)^¶^ | 29.9 (5.4) | 31.2 (5.1) | 30.5 (5.3) |
| G6PD status†  Normal  Heterozygous female  Deficient males and females | 89 (70.1)  12 (9.4)  26 (20.5) | 92 (70.8)  9 (6.9)  29 (22.3) | 181 (70.4)  21 (8.2)  55 (21.4) |
| Sickle cell status† |  |  |  |
| Normal (HbAA) | 114 (89.8) | 113 (86.9) | 227 (88.3) |
| Trait (HbAS) | 12 (9.4) | 15 (11.5) | 27 (10.5) |
| Disease (HbSS) | 1 (0.8) | 2 (1.5) | 3 (1.2) |
| Thalassemia status† |  |  |  |
| Normal (αα/αα) | 56 (44.1) | 58 (45.0) | 114 (44.5) |
| Heterozygous (-α/αα) | 59 (46.5) | 60 (46.5) | 119 (46.5) |
| Homozygous (-α/-α) | 12 (9.4) | 11 (8.5) | 23 (9.0) |
| Primaquine dose (mg/kg)^* §‡^ | 0.21 (0.17, 0.25) | 0.20 (0.16, 0.24) | 0.21 (0.16, 0.25) |
|  | [0.11, 0.34] | [0.10, 0.41] | [0.10, 0.41] |

* Indicates unit in median (IQR)

† Indicates n (%)

^#^ Indicates geometric mean [range]

^§^ Indicates range [Minimum, Maximum]

^¶^ Indicates mean (SD)

^‡^ Indicates the placebo dose

# Table S3. Factors associated with nadir haemoglobin, including mg/kg dose of primaquine and CYP2D6 activity score (N=258).

| **Variables** | **Univariate analysis** | | **Multivariable analysis** | |
| --- | --- | --- | --- | --- |
|  | **Slope (95% CI)** | **p-value** | **Slope (95% CI)** | **p-value** |
| Age (years) | 0.20 (0.14, 0.26) | <0.001 | 0.08 (0.02, 0.14) | 0.005 |
| Sex  Male (reference) |  |  |  |  |
| Female | 0.20 (-0.21, 0.60) | 0.335 | -0.02 (-0.26, 0.21) | 0.838 |
| Length of illness before treatment (days) | -0.25 (-0.40, -0.10) | <0.001 | -0.02 (-0.11, 0.07) | 0.600 |
| Mid-upper arm circumference (cm) | 0.29 (0.19, 0.38) | <0.001 | -0.03 (-0.12, 0.05) | 0.438 |
| Baseline haemoglobin (g/dL) | 0.77 (0.70, 0.84) | <0.001 | 0.78 (0.70, 0.85) | <0.001 |
| Splenomegaly  Normal (reference) | -0.58 (-1.05, -0.10) | 0.018 | 0.33 (0.05, 0.61) | 0.022 |
| Hepatomegaly  Normal (reference) | -0.36 (-1.44, 0.72) | 0.511 | NA | NA |
| Sickle cell status  Normal (HbAA) (reference) |  |  |  |  |
| Trait (HbAS) | -0.09 (-0.73, 0.55) | 0.789 | NA | NA |
| Disease (HbSS)* | -0.88 (-2.72, 0.96) | 0.348 | NA | NA |
| Thalassemia status  Normal (αα/αα) (reference) |  |  |  |  |
| Heterozygous (-α/αα) | -0.27 (-0.67, 0.14) | 0.202 | 0.20 (-0.04, 0.44) | 0.109 |
| Homozygous (-α/-α) | -1.11 (-1.82, -0.40) | 0.002 | 0.09 (-0.33, 0.52) | 0.673 |
| G6PD  Normal (reference) |  |  |  |  |
| Heterozygous females | 0.05 (-0.68, 0.78) | 0.901 | NA | NA |
| Deficient males and females | -0.39 (-0.87, 0.10) | 0.121 | NA | NA |
| Log of baseline parasitaemia | -0.12 (-0.20, -0.04) | 0.004 | -0.11 (-0.15, -0.07) | <0.001 |
| Mg/kg of primaquine | 2.01 (-1.67, 5.69) | 0.283 | NA | NA |
| Activity Score | -0.10 (-0.53, 0.33) | 0.646 | NA | NA |

* N=3 patients

Table S3 multivariable adjusted R-squared: 0.7518

# Table S4. Factors associated with fractional fall (%) in haemoglobin, including mg/kg dose of primaquine and CYP2D6 activity score (N=258).

| **Variables** | **Univariate analysis** | | **Multivariable analysis** | |
| --- | --- | --- | --- | --- |
|  | **Slope (95% CI)** | **P-values** | **Slope (95% CI)** | **P-values** |
| Age (years) | -0.51 (-0.87, -0.15) | 0.006 | -0.64 (-1.02, -0.26) | 0.001 |
| Sex  Male (reference) |  |  |  |  |
| Female | -0.08 (-2.35, 2.19) | 0.946 | 0.24 (-1.98, 2.46) | 0.832 |
| Length of illness before treatment (days) | 0.35 (-0.50, 1.19) | 0.421 | NA | NA |
| Mid-upper arm circumference (cm) | -0.37 (-0.95, 0.20) | 0.205 | NA | NA |
| Baseline haemoglobin (g/dL) | 0.85 (0.20, 1.50) | 0.011 | 0.86 (0.18, 1.54) | 0.013 |
| Splenomegaly  Normal (reference) | -4.66 (-7.30, -2.01) | 0.001 | -3.85 (-6.43, -1.28) | 0.004 |
| Hepatomegaly  Normal (reference) | -4.14 (-10.16, 1.88) | 0.177 | NA | NA |
| Sickle cell status  Normal (HbAA) (reference) |  |  |  |  |
| Trait (HbAS) | -1.65 (-5.27, 1.97) | 0.371 | NA | NA |
| Disease (HbSS)* | -7.19 (-17.52, 3.14) | 0.172 | NA | NA |
| Thalassemia status  Normal (αα/αα) (reference) |  |  |  |  |
| Heterozygous (-α/αα) | -1.55 (-3.88, 0.78) | 0.191 | NA | NA |
| Homozygous (-α/-α) | -3.40 (-7.47, 0.66) | 0.100 | NA | NA |
| G6PD  Normal (reference) |  |  |  |  |
| Heterozygous females | 0.75 (-3.36, 4.85) | 0.721 | NA | NA |
| Deficient males and females | -1.45 (-4.20, 1.29) | 0.297 | NA | NA |
| Log of baseline parasitaemia | 1.05 (0.64, 1.46) | <0.001 | 1.07 (0.68, 1.45) | <0.001 |
| Mg/kg of primaquine | -12.84 (-33.42, 7.75) | 0.221 | NA | NA |
| Activity Score | 0.76 (-1.49, 3.01) | 0.507 | NA | NA |

* N=3 patients

Table S4 multivariable adjusted R-squared: 0.2069

# Table S5. Factors associated with haemoglobin concentration at day 42 including mg/kg dose of primaquine and CYP2D6 activity score (N=258).

| **Variables** | **Univariate analysis** | | **Multivariable analysis** | |
| --- | --- | --- | --- | --- |
|  | **Slope (95% CI)** | **P-value** | **Slope (95% CI)** | **P-value** |
| Age (years) | 0.19 (0.14, 0.23) | <0.001 | 0.13 (0.09, 0.18) | <0.001 |
| Sex  Male (reference) |  |  |  |  |
| Females | 0.01 (-0.31, 0.31) | 0.973 | -0.06 (-0.32, 0.19) | 0.625 |
| Length of illness before treatment (days) | -0.09 (-0.21, 0.02) | 0.111 | NA | NA |
| Fractional fall (%) in haemoglobin | 0.36 (0.27, 0.44) | <0.001 | 0.21 (0.07, 0.35) | 0.003 |
| Baseline haemoglobin (g/dL) | 0.30 (0.22, 0.38) | <0.001 | 0.02 (-0.11, 0.15) | 0.720 |
| Splenomegaly  Normal (reference) | -0.04 (-0.41, 0.33) | 0.827 | NA | NA |
| Sickle cell status  Normal (HbAA) (reference) |  |  |  |  |
| Trait (HbAS) | -0.42 (-0.92, 0.07) | 0.094 | -0.35 (-0.76, 0.06) | 0.094 |
| Disease (HbSS) * | -3.33 (-4.96, -1.70) | <0.001 | -2.81 (-4.19, -1.44) | <0.001 |
| Thalassemia status  Normal (αα/αα) (reference) |  |  |  |  |
| Heterozygous (-α/αα) | -0.44 (-0.75, -0.12) | 0.007 | -0.24 (-0.50, 0.02) | 0.073 |
| Homozygous (-α/-α) | -0.63 (-1.18, -0.08) | 0.024 | -0.27 (-0.73, 0.20) | 0.253 |
| G6PD status  Normal (reference) |  |  |  |  |
| Heterozygous females | -0.17 (-0.73, 0.39) | 0.548 | NA | NA |
| Deficient males and females | 0.11 (-0.26, 0.48) | 0.563 | NA | NA |
| Log of baseline parasitaemia | -0.03 (-0.09, 0.03) | 0.290 | NA | NA |
| Treatment failure  ACPR (reference) | -0.66 (-1.19, -0.12) | 0.017 | -0.30 (-0.74, 0.14) | 0.183 |
| Mg/kg of primaquine | 2.24 (-0.60, 5.08) | 0.122 | NA | NA |
| Activity Score | 0.10 (-0.22, 0.41) | 0.556 | NA | NA |

* N=3 patients

Table S5 multivariable adjusted R-squared: 0.3673

# Table S6. Factors associated with haemoglobin recovery on Day 42, including mg/kg dose of primaquine and CYP2D6 activity score (N=258).

| **Variables** | **Univariate logistic regression** | | **Multivariable logistic regression** | |
| --- | --- | --- | --- | --- |
|  | **Odds ratio (95% CI)** | **P-values** | **Adjusted odds ratio (95% CI)** | **P-values** |
| Age (years) | 1.04 (0.94, 1.16) | 0.410 | 1.28 (1.09, 1.50) | 0.003 |
| Sex  Male (reference) |  |  |  |  |
| Females | 0.86 (0.46, 1.62) | 0.643 | 1.13 (0.53, 2.44) | 0.747 |
| Length of illness before treatment (days) | 1.27 (0.96, 1.66) | 0.089 | NA | NA |
| Fractional fall (%) in haemoglobin | 0.73 (0.59, 0.91) | 0.004 | 1.79 (1.21, 2.66) | 0.004 |
| Baseline haemoglobin (g/dL) | 0.43 (0.32, 0.58) | <0.001 | 0.20 (0.12, 0.34) | <0.001 |
| Splenomegaly  Normal (Reference) | 4.08 (1.40, 11.90) | 0.010 | 0.99 (0.28, 3.58) | 0.994 |
| Sickle cell status  Normal (HbAA) (reference) |  |  | NA | NA |
| Trait (HbAS) | 0.99 (0.35, 2.79) | 0.983 | NA | NA |
| Disease (HbSS) * | 0.26 (0.02, 4.24) | 0.344 | NA | NA |
| Thalassemia status  Normal (αα/αα) (reference) |  |  | NA | NA |
| Heterozygous (-α/αα) | 1.48 (0.79, 2.79) | 0.224 | NA | NA |
| Homozygous (-α/-α) | 7.44 (0.97, 57.93) | 0.055 | NA | NA |
| G6PD  Normal (reference) |  |  |  |  |
| Heterozygous females | 0.89 (0.30, 2.59) | 0.826 | NA | NA |
| Deficient males and females | 1.94 (0.81, 4.64) | 0.136 | NA | NA |
| Log of baseline parasitaemia | 0.94 (0.82, 1.09) | 0.422 | NA | NA |
| Treatment failure  ACPR (reference) | 0.63 (0.23, 1.71) | 0.363 | NA | NA |
| Mg/kg of primaquine | 62.79 (0.15, 26609.34) | 0.180 | NA | NA |
| Activity Score | 1.16 (0.61, 2.21) | 0.645 | NA | NA |

* N=3 patients

Table S6 Akaike Information Criterion (AIC): 188.66

# Table S7. Factors associated with malaria attributable fraction, including mg/kg dose of primaquine and CYP2D6 activity score (N=258).

| **Variables** | **Univariate analysis** | | **Multivariable analysis** | |
| --- | --- | --- | --- | --- |
|  | **Slope (95% CI)** | **p-value** | **Slope (95% CI)** | **p-value** |
| Age (years) | -0.02 (-0.08, 0.05) | 0.608 | 0.17 (0.10, 0.24) | <0.001 |
| Sex  Male (reference)  Female | -0.23 (-0.61, 0.15) | 0.236 | 0.01 (-0.27, 0.28) | 0.9695 |
| Length of illness before treatment (days) | 0.16 (0.01, 0.30) | 0.033 | 0.07 (-0.04, 0.17) | 0.225 |
| Mid-upper arm circumference (cm) | -0.11 (-0.21, -0.02) | 0.020 | -0.13 (-0.23, -0.03) | 0.012 |
| Fractional fall (%) in haemoglobin | -0.64 (-0.73, -0.56) | <0.001 | -0.86 (-1.02, -0.70) | <0.001 |
| Baseline haemoglobin (g/dL) | -0.47 (-0.56, -0.38) | <0.001 | 0.15 (0.001, 0.30) | 0.049 |
| Splenomegaly  Normal (reference) | 0.61 (0.17, 1.05) | 0.007 | 0.10 (-0.22, 0.43) | 0.526 |
| Hepatomegaly  Normal (reference) | 0.30 (-0.69, 1.30) | 0.547 | NA | NA |
| Sickle cell status  Normal (HbAA) (reference) |  |  |  |  |
| Trait (HbAS) | -0.40 (-1.02, 0.24) | 0.222 | NA | NA |
| Disease (HbSS) * | -1.26 (-3.33, 0.81) | 0.232 | NA | NA |
| Thalassemia status  Normal (αα/αα) (reference) |  |  |  |  |
| Heterozygous (-α/αα) | -0.15 (-0.54, 0.24) | 0.463 | NA | NA |
| Homozygous (-α/-α) | 0.41 (-0.27, 1.09) | 0.230 | NA | NA |
| G6PD  Normal (reference) |  |  | NA | NA |
| Heterozygous females | -0.09 (-0.77, 0.60) | 0.807 | NA | NA |
| Deficient males and females | 0.45 (-0.004, 0.91) | 0.052 | NA | NA |
| Log of baseline parasitaemia | 0.10 (0.02, 0.17) | 0.013 | -0.005 (-0.06, 0.05) | 0.853 |
| Mg/kg of primaquine | -0.37 (-3.87, 3.14) | 0.836 | NA | NA |
| Activity Score | 0.13 (-0.27, 0.54) | 0.516 | NA | NA |

* N=3 patients

Table S7 multivariable adjusted R-squared: 0.5913

# Table S8. Factors associated with time to haemoglobin recovery, including mg/kg dose of primaquine and CYP2D6 activity score (N=258).

| **Variables** | **Univariate analysis** | | **Multivariable analysis** | |
| --- | --- | --- | --- | --- |
|  | **HR (95% CI)** | **p-value** | **Adjusted HR (95% CI)** | **p-value** |
| Age (years) | 0.98 (0.94, 1.01) | 0.297 | 1.05 (1.00, 1.10) | 0.041 |
| Sex  Male (reference)  Female | 1.02 (0.78, 1.33) | 0.880 | NA | NA |
| Length of illness before treatment (days) | 1.05 (0.96, 1.15) | 0.261 | NA | NA |
| Mid-upper arm circumference (cm) | 0.96 (0.90, 1.02) | 0.176 | NA | NA |
| Fractional fall (%) in haemoglobin | 0.93 (0.86, 1.00) | 0.072 | NA | NA |
| Baseline haemoglobin (g/dL) | 0.77 (0.71, 0.83) | <0.001 | 0.73 (0.66, 0.79) | <0.001 |
| Splenomegaly  Normal (reference) | 1.35 (0.99, 1.84) | 0.059 | NA | NA |
| Hepatomegaly  Normal (reference) | 1.48 (0.76, 2.89) | 0.248 | NA | NA |
| Sickle cell status  Normal (HbAA) (reference) |  |  |  |  |
| Trait (HbAS) | 1.21 (0.79, 1.84) | 0.384 | NA | NA |
| Disease (HbSS)* | 0.59 (0.15, 2.37) | 0.457 | NA | NA |
| Thalassemia status  Normal (αα/αα) (reference) |  |  |  |  |
| Heterozygous (-α/αα) | 1.01 (0.77, 1.33) | 0.907 | 0.88 (0.64, 1.19) | 0.425 |
| Homozygous (-α/-α) | 1.72 (1.09, 2.73) | 0.019 | 1.11 (0.67, 1.86) | 0.685 |
| G6PD  Normal (reference) |  |  |  |  |
| Heterozygous females | 0.85 (0.52, 1.37) | 0.503 | NA | NA |
| Deficient males and females | 1.15 (0.52, 1.58) | 0.376 | NA | NA |
| Log of baseline parasitaemia | 0.90 (0.86, 0.95) | <0.001 | 0.89 (0.84, 0.93) | <0.001 |
| Treatment failure  ACPR (reference) | 1.04 (0.64, 1.68) | 0.880 | NA | NA |
| Mg/kg of primaquine | 0.84 (0.07, 9.51) | 0.888 | NA | NA |
| Activity Score | 1.18 (0.90, 1.55) | 0.236 | NA | NA |

* N=3 patients

Table S8 Akaike Information Criterion (AIC): 1703.44

# Table S9. Factors associated with nadir haemoglobin, including primaquine AUC_0-t_ (N=258).

| **Variables** | **Univariate analysis** | | **Multivariable analysis** | |
| --- | --- | --- | --- | --- |
|  | **Slope (95% CI)** | **p-value** | **Slope (95% CI)** | **p-value** |
| Age (years) | 0.20 (0.14, 0.26) | <0.001 | 0.08 (0.02, 0.14) | 0.005 |
| Sex  Male (reference) |  |  |  |  |
| Female | 0.20 (-0.21, 0.60) | 0.335 | -0.02 (-0.26, 0.21) | 0.838 |
| Length of illness before treatment (days) | -0.25 (-0.40, -0.10) | <0.001 | -0.02 (-0.11, 0.07) | 0.600 |
| Mid-upper arm circumference (cm) | 0.29 (0.19, 0.38) | <0.001 | -0.03 (-0.12, 0.05) | 0.438 |
| Baseline haemoglobin (g/dL) | 0.77 (0.70, 0.84) | <0.001 | 0.78 (0.70, 0.85) | <0.001 |
| Splenomegaly  Normal (reference) | -0.58 (-1.05 -0.10) | 0.018 | 0.33 (0.05, 0.61) | 0.022 |
| Hepatomegaly  Normal (reference) | -0.36 (-1.44, 0.72) | 0.511 | NA | NA |
| Sickle cell status  Normal (HbAA) (reference) |  |  |  |  |
| Trait (HbAS) | -0.09 (-0.73, 0.55) | 0.789 | NA | NA |
| Disease (HbSS) * | -0.88 (-2.72, 0.96) | 0.348 | NA | NA |
| Thalassemia status  Normal (αα/αα) (reference) |  |  |  |  |
| Heterozygous (-α/αα) | -0.27 (-0.67, 0.14) | 0.202 | 0.20 (-0.04, 0.44) | 0.109 |
| Homozygous (-α/-α) | -1.11 (-1.82, -0.40) | 0.002 | 0.09 (-0.33, 0.52) | 0.673 |
| G6PD  Normal (reference) |  |  |  |  |
| Heterozygous females | 0.05 (-0.68, 0.78) | 0.901 | NA | NA |
| Deficient males and females | -0.39 (-0.87, 0.10) | 0.121 | NA | NA |
| Log of baseline parasitaemia | -0.12 (-0.20, -0.04) | 0.004 | -0.11 (-0.15, -0.07) | <0.001 |
| AUC _0-last_ (ng*h/mL) of primaquine | -0.0004 (-0.001, 0.0001) | 0.124 | NA | NA |

* N=3 patients

Table S9 multivariable adjusted R-squared: 0.7518

# Table S10. Factors associated with fractional fall (%) in haemoglobin, including primaquine AUC_0-t_ (N=258).

| **Variables** | **Univariate analysis** | | **Multivariable analysis** | |
| --- | --- | --- | --- | --- |
|  | **Slope (95% CI)** | **P-values** | **Slope (95% CI)** | **P-values** |
| Age (years) | -0.51 (-0.87, -0.15) | 0.006 | -0.64 (-1.02, -0.26) | 0.001 |
| Sex  Male (reference)  Female | -0.08 (-2.35, 2.19) | 0.946 | 0.24 (-1.98, 2.46) | 0.832 |
| Length of illness before treatment (days) | 0.35 (-0.50, 1.19) | 0.421 | NA | NA |
| Mid-upper arm circumference (cm) | -0.37 (-0.95, 0.20) | 0.205 | NA | NA |
| Baseline haemoglobin (g/dL) | 0.85 (0.20, 1.50) | 0.011 | 0.86 (0.18, 1.54) | 0.013 |
| Splenomegaly  Normal (reference) | -4.66 (-7.30, -2.01) | 0.001 | -3.85 (-6.43, -1.28) | 0.004 |
| Hepatomegaly  Normal (reference) | -4.14 (-10.16, 1.88) | 0.177 | NA | NA |
| Sickle cell status  Normal (HbAA) (reference) |  |  |  |  |
| Trait (HbAS) | -1.65 (-5.27, 1.97) | 0.371 | NA | NA |
| Disease (HbSS) * | -7.19 (-17.52, 3.14) | 0.172 | NA | NA |
| Thalassemia status  Normal (αα/αα) (reference) |  |  |  |  |
| Heterozygous (-α/αα) | -1.55 (-3.88, 0.78) | 0.191 | NA | NA |
| Homozygous (-α/-α) | -3.40 (-7.47, 0.66) | 0.100 | NA | NA |
| G6PD  Normal (reference) |  |  |  |  |
| Heterozygous females | 0.75 (-3.36, 4.85) | 0.721 | NA | NA |
| Deficient males and females | -1.45 (-4.20, 1.29) | 0.297 | NA | NA |
| Log of baseline parasitaemia | 1.05 (0.64, 1.46) | <0.001 | 1.07 (0.68, 1.45) | <0.001 |
| AUC _0-last_ (ng*h/mL) of primaquine | 0.001 (-0.002, 0.004) | 0.496 | NA | NA |

* N=3 patients

Table S10 multivariable adjusted R-squared: 0.2069

# Table S11. Factors associated with haemoglobin concentration at day 42, including primaquine AUC_0-t_ (N=258).

| **Variables** | **Univariate analysis** | | **Multivariable analysis** | |
| --- | --- | --- | --- | --- |
|  | **Slope (95% CI)** | **P-value** | **Slope (95% CI)** | **P-value** |
| Age (years) | 0.19 (0.14, 0.23) | <0.001 | 0.13 (0.09, 0.18) | <0.001 |
| Sex  Male (reference) |  |  |  |  |
| Females | 0.01 (-0.31, 0.31) | 0.973 | -0.06 (-0.32, 0.19) | 0.625 |
| Length of illness before treatment (days) | -0.09 (-0.21, 0.02) | 0.111 | NA | NA |
| Fractional fall (%) in haemoglobin | 0.36 (0.27, 0.44) | <0.001 | 0.21 (0.07, 0.35) | 0.003 |
| Baseline haemoglobin (g/dL) | 0.30 (0.22, 0.38) | <0.001 | 0.02 (-0.11, 0.15) | 0.720 |
| Splenomegaly  Normal (reference) | -0.04 (-0.41, 0.33) | 0.827 | NA | NA |
| Sickle cell status  Normal (HbAA) (reference) |  |  |  |  |
| Trait (HbAS) | -0.42 (-0.92, 0.07) | 0.094 | -0.35 (-0.76, 0.06) | 0.094 |
| Disease (HbSS) * | -3.33 (-4.96, -1.70) | <0.001 | -2.81 (-4.19, -1.44) | <0.001 |
| Thalassemia status  Normal (αα/αα) (reference) |  |  |  |  |
| Heterozygous (-α/αα) | -0.44 (-0.75, -0.12) | 0.007 | -0.24 (-0.50, 0.02) | 0.073 |
| Homozygous (-α/-α) | -0.63 (-1.18, -0.08) | 0.024 | -0.27 (-0.73, 0.20) | 0.253 |
| G6PD  Normal (reference) |  |  |  |  |
| Heterozygous females | -0.17 (-0.73, 0.39) | 0.548 | NA | NA |
| Deficient males and females | 0.11 (-0.26, 0.48) | 0.563 | NA | NA |
| Log of baseline parasitaemia | -0.03 (-0.09, 0.03) | 0.290 | NA | NA |
| Treatment failure  ACPR (reference) | -0.66 (-1.19, -0.12) | 0.017 | -0.30 (-0.74, 0.14) | 0.183 |
| AUC _0-last_ (ng*h/mL) of primaquine | 0.0001 (-0.0002, 0.0004) | 0.615 | NA | NA |

* N=3 patients

Table S11 multivariable adjusted R-squared: 0.3673

# Table S12. Factors associated with haemoglobin recovery at day 42, including primaquine AUC_0-t_ (N=258).

| **Variables** | **Univariate logistic regression** | | **Multivariable logistic regression** | |
| --- | --- | --- | --- | --- |
|  | **Odds ratio (95% CI)** | **P-values** | **Adjusted odds ratio (95% CI)** | **P-values** |
| Age (years) | 1.04 (0.94, 1.16) | 0.410 | 1.28 (1.09, 1.50) | 0.003 |
| Sex  Male (reference)  Females | 0.86 (0.46, 1.62) | 0.643 | 1.13 (0.53, 2.44) | 0.747 |
| Length of illness before treatment (days) | 1.27 (0.96, 1.66) | 0.089 | NA | NA |
| Fractional fall (%) in haemoglobin | 0.73 (0.59, 0.91) | 0.004 | 1.79 (1.21, 2.66) | 0.004 |
| Baseline haemoglobin (g/dL) | 0.43 (0.32, 0.58) | <0.001 | 0.20 (0.12, 0.33) | <0.001 |
| Splenomegaly  Normal (Reference) | 4.08 (1.40, 11.90) | 0.010 | 0.99 (0.28, 3.58) | 0.994 |
| Sickle cell status  Normal (HbAA) (reference) |  |  |  |  |
| Trait (HbAS) | 0.99 (0.35, 2.79) | 0.983 | NA | NA |
| Disease (HbSS) * | 0.26 (0.02, 4.24) | 0.344 | NA | NA |
| Thalassemia status  Normal (αα/αα) (reference) |  |  |  |  |
| Heterozygous (-α/αα) | 1.48 (0.79, 2.79) | 0.224 | NA | NA |
| Homozygous (-α/-α) | 7.44 (0.97, 57.93) | 0.055 | NA | NA |
| G6PD  Normal (reference) |  |  |  |  |
| Heterozygous females | 0.89 (0.30, 2.59) | 0.826 | NA | NA |
| Deficient males and females | 1.94 (0.81, 4.64) | 0.136 | NA | NA |
| Log of baseline parasitaemia | 0.94 (0.82, 1.09) | 0.422 | NA | NA |
| Treatment failure  ACPR (reference) | 0.63 (0.23, 1.71) | 0.363 | NA | NA |
| AUC _0-last_ (ng*h/mL) of primaquine | 1.00 (1.00, 1.00) | 0.404 | NA | NA |

* N=3 patients

Table S12 Akaike Information Criterion (AIC): 188.66

# Table S13. Factors associated with malaria attributable fraction, including primaquine AUC_0-t_ (N=258).

| **Variables** | **Univariate analysis** | | **Multivariable analysis** | |
| --- | --- | --- | --- | --- |
|  | **Slope (95% CI)** | **p-value** | **Slope (95% CI)** | **p-value** |
| Age (years) | -0.02 (-0.08, 0.05) | 0.608 | 0.18 (0.11, 0.25) | <0.001 |
| Sex  Male (reference)  Female | -0.23 (-0.61, 0.15) | 0.236 | -0.002 (-0.28, 0.28) | 0.989 |
| Length of illness before treatment (days) | 0.16 (0.01, 0.30) | 0.033 | 0.06 (-0.05, 0.17) | 0.257 |
| Mid-upper arm circumference (cm) | -0.11 (-0.21, -0.02) | 0.020 | -0.13 (-0.24, -0.03) | 0.011 |
| Fractional fall (%) in haemoglobin | -0.64 (-0.73, -0.56) | <0.001 | -0.87 (-1.04, -0.69) | <0.001 |
| Baseline haemoglobin (g/dL) | -0.47 (-0.56, -0.38) | <0.001 | 0.15 (-0.01, 0.31) | 0.074 |
| Splenomegaly  Normal (reference) | 0.61 (0.17, 1.05) | 0.007 | 0.09 (-0.24, 0.42) | 0.609 |
| Hepatomegaly  Normal (reference) | 0.30 (-0.69, 1.30) | 0.547 | NA | NA |
| Sickle cell status  Normal (HbAA) (reference) |  |  |  |  |
| Trait (HbAS) | -0.40 (-1.02, 0.24) | 0.222 | NA | NA |
| Disease (HbSS) * | -1.26 (-3.33, 0.81) | 0.232 | NA | NA |
| Thalassemia status  Normal (αα/αα) (reference) |  |  |  |  |
| Heterozygous (-α/αα) | -0.15 (-0.54, 0.24) | 0.463 | NA | NA |
| Homozygous (-α/-α) | 0.41 (-0.27, 1.09) | 0.230 | NA | NA |
| G6PD  Normal (reference) |  |  |  |  |
| Heterozygous females | -0.09 (-0.77, 0.60) | 0.807 | NA | NA |
| Deficient males and females | 0.45 (-0.004, 0.91) | 0.052 | NA | NA |
| Log of baseline parasitaemia | 0.10 (0.02, 0.17) | 0.013 | -0.006 (-0.06, 0.04) | 0.821 |
| AUC _0-last_ (ng*h/mL) of primaquine | 0.001 (0.00004, 0.001) | 0.031 | -0.0002 (-0.001, 0.0001) | 0.261 |

* N=3 patients

Table S13 multivariable adjusted R-squared: 0.5899

# Table S14. Factors associated with time to haemoglobin recovery, including primaquine AUC_0-t_ (N=258).

| **Variables** | **Univariate analysis** | | **Multivariable analysis** | |
| --- | --- | --- | --- | --- |
|  | **HR (95% CI)** | **p-value** | **Adjusted HR (95% CI)** | **p-value** |
| Age (years) | 0.98 (0.94, 1.01) | 0.297 | 1.05 (1.00, 1.10) | 0.041 |
| Sex  Male (reference)  Female | 1.02 (0.78, 1.33) | 0.880 | NA | NA |
| Length of illness before treatment (days) | 1.05 (0.96, 1.15) | 0.261 | NA | NA |
| Mid-upper arm circumference (cm) | 0.96 (0.90, 1.02) | 0.176 | NA | NA |
| Fractional fall (%) in haemoglobin | 0.93 (0.86, 1.00) | 0.072 | NA | NA |
| Baseline haemoglobin (g/dL) | 0.77 (0.71, 0.83) | <0.001 | 0.73 (0.66, 0.79) | <0.001 |
| Splenomegaly  Normal (reference) | 1.35 (0.99, 1.84) | 0.059 | NA | NA |
| Hepatomegaly  Normal (reference) | 1.48 (0.76, 2.89) | 0.248 | NA | NA |
| Sickle cell status  Normal (HbAA) (reference) |  |  |  |  |
| Trait (HbAS) | 1.21 (0.79, 1.84) | 0.384 | NA | NA |
| Disease (HbSS) * | 0.59 (0.15, 2.37) | 0.457 | NA | NA |
| Thalassemia status  Normal (αα/αα) (reference) |  |  |  |  |
| Heterozygous (-α/αα) | 1.01 (0.77, 1.33) | 0.907 | 0.88 (0.64, 1.20) | 0.425 |
| Homozygous (-α/-α) | 1.72 (1.09, 2.73) | 0.019 | 1.11 (0.67, 1.86) | 0.671 |
| G6PD  Normal (reference) |  |  |  |  |
| Heterozygous females | 0.85 (0.52, 1.37) | 0.503 | NA | NA |
| Deficient males and females | 1.15 (0.52, 1.58) | 0.376 | NA | NA |
| Log of baseline parasitaemia | 0.90 (0.86, 0.95) | <0.001 | 0.89 (0.84, 0.93) | <0.001 |
| Treatment failure  ACPR (reference) | 1.04 (0.64, 1.68) | 0.880 | NA | NA |
| AUC 0-last (ng*h/mL) of primaquine | 1.00 (1.00, 1.00) | 0.517 | NA | NA |

* N=3 patients

Table S14 Akaike Information Criterion (AIC): 1703.44

# Table S15. Factors associated with nadir haemoglobin, including primaquine *C*_max_ (N=258).

| **Variables** | **Univariate analysis** | | **Multivariable analysis** | |
| --- | --- | --- | --- | --- |
|  | **Slope (95% CI)** | **p-value** | **Slope (95% CI)** | **p-value** |
| Age (years) | 0.20 (0.14, 0.26) | <0.001 | 0.08 (0.02, 0.14) | 0.005 |
| Sex  Male (reference) |  |  |  |  |
| Female | 0.20 (-0.21, 0.60) | 0.335 | -0.02 (-0.26, 0.21) | 0.838 |
| Length of illness before treatment (days) | -0.25 (-0.40, -0.10) | <0.001 | -0.02 (-0.11, 0.07) | 0.600 |
| Mid-upper arm circumference (cm) | 0.29 (0.19, 0.38) | <0.001 | -0.03 (-0.12, 0.05) | 0.438 |
| Baseline haemoglobin (g/dL) | 0.77 (0.70, 0.84) | <0.001 | 0.78 (0.70, 0.85) | <0.001 |
| Splenomegaly  Normal (reference) | -0.58 (-1.05 -0.10) | 0.018 | 0.33 (0.05, 0.61) | 0.022 |
| Hepatomegaly  Normal (reference) | -0.36 (-1.44, 0.72) | 0.511 | NA | NA |
| Sickle cell status  Normal (HbAA) (reference) |  |  |  |  |
| Trait (HbAS) | -0.09 (-0.73, 0.55) | 0.789 | NA | NA |
| Disease (HbSS) * | -0.88 (-2.72, 0.96) | 0.348 | NA | NA |
| Thalassemia status  Normal (αα/αα) (reference) |  |  |  |  |
| Heterozygous (-α/αα) | -0.27 (-0.67, 0.14) | 0.202 | 0.20 (-0.04, 0.44) | 0.109 |
| Homozygous (-α/-α) | -1.11 (-1.82, -0.40) | 0.002 | 0.09 (-0.33, 0.52) | 0.673 |
| G6PD  Normal (reference) |  |  |  |  |
| Heterozygous females | 0.05 (-0.68, 0.78) | 0.901 | NA | NA |
| Deficient males and females | -0.39 (-0.87, 0.10) | 0.121 | NA | NA |
| Log of baseline parasitaemia | -0.12 (-0.20, -0.04) | 0.004 | -0.11 (-0.15, -0.07) | <0.001 |
| *C*max (ng/mL) | -0.002 (-0.01, 0.001) | 0.266 | NA | NA |

* N=3 patients

Table S15 multivariable adjusted R-squared: 0.7518

# Table S16. Factors associated with fractional Fall (%) in haemoglobin, including primaquine *C*_max_ (N=258).

| **Variables** | **Univariate analysis** | | **Multivariable analysis** | |
| --- | --- | --- | --- | --- |
|  | **Slope (95% CI)** | **P-values** | **Slope (95% CI)** | **P-values** |
| Age (years) | -0.51 (-0.87, -0.15) | 0.006 | -0.64 (-1.02, -0.26) | 0.001 |
| Sex  Male (reference)  Female | -0.08 (-2.35, 2.19) | 0.946 | 0.24 (-1.98, 2.46) | 0.832 |
| Length of illness before treatment (days) | 0.35 (-0.50, 1.19) | 0.421 | NA | NA |
| Mid-upper arm circumference (cm) | -0.37 (-0.95, 0.20) | 0.205 | NA | NA |
| Baseline haemoglobin (g/dL) | 0.85 (0.20, 1.50) | 0.011 | 0.86 (0.18, 1.54) | 0.013 |
| Splenomegaly  Normal (reference) | -4.66 (-7.30, -2.01) | 0.001 | -3.85 (-6.43, -1.28) | 0.004 |
| Hepatomegaly  Normal (reference) | -4.14 (-10.16, 1.88) | 0.177 | NA | NA |
| Sickle cell status  Normal (HbAA) (reference) |  |  |  |  |
| Trait (HbAS) | -1.65 (-5.27, 1.97) | 0.371 | NA | NA |
| Disease (HbSS) * | -7.19 (-17.52, 3.14) | 0.172 | NA | NA |
| Thalassemia status  Normal (αα/αα) (reference) |  |  |  |  |
| Heterozygous (-α/αα) | -1.55 (-3.88, 0.78) | 0.191 | NA | NA |
| Homozygous (-α/-α) | -3.40 (-7.47, 0.66) | 0.100 | NA | NA |
| G6PD  Normal (reference) |  |  |  |  |
| Heterozygous females | 0.75 (-3.36, 4.85) | 0.721 | NA | NA |
| Deficient males and females | -1.45 (-4.20, 1.29) | 0.297 | NA | NA |
| Log of baseline parasitaemia | 1.05 (0.64, 1.46) | <0.001 | 1.07 (0.68, 1.45) | <0.001 |
| *C*max (ng/mL) | -0.001 (-0.02, 0.02) | 0.886 | NA | NA |

* N=3 patients

Table S16 multivariable adjusted R-squared: 0.2069

# Table S17. Factors associated with haemoglobin concentration at day 42, including primaquine *C*_max_ (N=258).

| **Variables** | **Univariate analysis** | | **Multivariable analysis** | |
| --- | --- | --- | --- | --- |
|  | **Slope (95% CI)** | **P-value** | **Slope (95% CI)** | **P-value** |
| Age (years) | 0.19 (0.14, 0.23) | <0.001 | 0.13 (0.09, 0.18) | <0.001 |
| Sex  Male (reference) |  |  |  |  |
| Females | 0.01 (-0.31, 0.31) | 0.973 | -0.06 (-0.32, 0.19) | 0.625 |
| Length of illness before treatment (days) | -0.09 (-0.21, 0.02) | 0.111 | NA | NA |
| Fractional fall (%) in haemoglobin | 0.36 (0.27, 0.44) | <0.001 | 0.21 (0.07, 0.35) | 0.003 |
| Baseline haemoglobin (g/dL) | 0.30 (0.22, 0.38) | <0.001 | 0.02 (-0.11, 0.15) | 0.720 |
| Splenomegaly  Normal (reference) | -0.04 (-0.41, 0.33) | 0.827 | NA | NA |
| Sickle cell status  Normal (HbAA) (reference) |  |  |  |  |
| Trait (HbAS) | -0.42 (-0.92, 0.07) | 0.094 | -0.35 (-0.76, 0.06) | 0.094 |
| Disease (HbSS) * | -3.33 (-4.96, -1.70) | <0.001 | -2.81 (-4.19, -1.44) | <0.001 |
| Thalassemia status  Normal (αα/αα) (reference) |  |  |  |  |
| Heterozygous (-α/αα) | -0.44 (-0.75, -0.12) | 0.007 | -0.24 (-0.50, 0.02) | 0.073 |
| Homozygous (-α/-α) | -0.63 (-1.18, -0.08) | 0.024 | -0.27 (-0.73, 0.20) | 0.253 |
| G6PD  Normal (reference) |  |  |  |  |
| Heterozygous females | -0.17 (-0.73, 0.39) | 0.548 | NA | NA |
| Deficient males and females | 0.11 (-0.26, 0.48) | 0.563 | NA | NA |
| Log of baseline parasitaemia | -0.03 (-0.09, 0.03) | 0.290 | NA | NA |
| Treatment failure  ACPR (reference) | -0.66 (-1.19, -0.12) | 0.017 | -0.30 (-0.74, 0.14) | 0.183 |
| *C*max (ng/mL) | 0.002 (-0.001, 0.004) | 0.161 | NA | NA |

* N=3 patients

Table S17 multivariable adjusted R-squared: 0.3673

# Table S18. Factors associated with haemoglobin recovery at day 42, including primaquine *C*_max_ (N=258).

| **Variables** | **Univariate logistic regression** | | **Multivariable logistic regression** | |
| --- | --- | --- | --- | --- |
|  | **Odds ratio (95% CI)** | **P-values** | **Adjusted odds ratio (95% CI)** | **P-values** |
| Age (years) | 1.04 (0.94, 1.16) | 0.410 | 1.28 (1.09, 1.50) | 0.003 |
| Sex  Male (reference)  Females | 0.86 (0.46, 1.62) | 0.643 | 1.13 (0.53, 2.44) | 0.747 |
| Length of illness before treatment (days) | 1.27 (0.96, 1.66) | 0.089 | NA | NA |
| Fractional fall (%) in haemoglobin | 0.73 (0.59, 0.91) | 0.004 | 1.79 (1.21, 2.66) | 0.004 |
| Baseline haemoglobin (g/dL) | 0.43 (0.32, 0.58) | <0.001 | 0.20 (0.12, 0.33) | <0.001 |
| Splenomegaly  Normal (Reference) | 4.08 (1.40, 11.90) | 0.010 | 0.99 (0.28, 3.58) | 0.994 |
| Sickle cell status  Normal (HbAA) (reference) |  |  |  |  |
| Trait (HbAS) | 0.99 (0.35, 2.79) | 0.983 | NA | NA |
| Disease (HbSS) * | 0.26 (0.02, 4.24) | 0.344 | NA | NA |
| Thalassemia status  Normal (αα/αα) (reference) |  |  |  |  |
| Heterozygous (-α/αα) | 1.48 (0.79, 2.79) | 0.224 | NA | NA |
| Homozygous (-α/-α) | 7.44 (0.97, 57.93) | 0.055 | NA | NA |
| G6PD  Normal (reference) |  |  |  |  |
| Heterozygous females | 0.89 (0.30, 2.59) | 0.826 | NA | NA |
| Deficient males and females | 1.94 (0.81, 4.64) | 0.136 | NA | NA |
| Log of baseline parasitaemia | 0.94 (0.82, 1.09) | 0.422 | NA | NA |
| Treatment failure  ACPR (reference) | 0.63 (0.23, 1.71) | 0.363 | NA | NA |
| *C*max (ng/mL) | 1.00 (1.00, 1.01) | 0.194 | NA | NA |

* N=3 patients

Table S18 Akaike Information Criterion (AIC): 188.66

# Table S19. Factors associated with malaria attributable fraction, including primaquine *C*_max_ (N=258).

| **Variables** | **Univariate analysis** | | **Multivariable analysis** | |
| --- | --- | --- | --- | --- |
|  | **Slope (95% CI)** | **p-value** | **Slope (95% CI)** | **p-value** |
| Age (years) | -0.02 (-0.08, 0.05) | 0.608 | 0.18 (0.11, 0.26) | <0.001 |
| Sex  Male (reference)  Female | -0.23 (-0.61, 0.15) | 0.236 | -0.01 (-0.29, 0.27) | 0.968 |
| Length of illness before treatment (days) | 0.16 (0.01, 0.30) | 0.033 | 0.06 (-0.05, 0.17) | 0.273 |
| Mid-upper arm circumference (cm) | -0.11 (-0.21, -0.02) | 0.020 | -0.13 (-0.24, -0.03) | 0.012 |
| Fractional fall (%) in haemoglobin | -0.64 (-0.73, -0.56) | <0.001 | -0.86 (-1.03, -0.68) | <0.001 |
| Baseline haemoglobin (g/dL) | -0.47 (-0.56, -0.38) | <0.001 | 0.14 (-0.02, 0.30) | 0.08 |
| Splenomegaly  Normal (reference) | 0.61 (0.17, 1.05) | 0.007 | 0.08 (-0.25, 0.42) | 0.613 |
| Hepatomegaly  Normal (reference) | 0.30 (-0.69, 1.30) | 0.547 | NA | NA |
| Sickle cell status  Normal (HbAA) (reference) |  |  |  |  |
| Trait (HbAS) | -0.40 (-1.02, 0.24) | 0.222 | NA | NA |
| Disease (HbSS) * | -1.26 (-3.33, 0.81) | 0.232 | NA | NA |
| Thalassemia status  Normal (αα/αα) (reference) |  |  |  |  |
| Heterozygous (-α/αα) | -0.15 (-0.54, 0.24) | 0.463 | NA | NA |
| Homozygous (-α/-α) | 0.41 (-0.27, 1.09) | 0.230 | NA | NA |
| G6PD  Normal (reference) |  |  |  |  |
| Heterozygous females | -0.09 (-0.77, 0.60) | 0.807 | NA | NA |
| Deficient males and females | 0.45 (-0.004, 0.91) | 0.052 | NA | NA |
| Log of baseline parasitaemia | 0.10 (0.02, 0.17) | 0.013 | -0.007 (-0.06, 0.04) | 0.780 |
| *C*max (ng/mL) | 0.003 (0.0001, 0.01) | 0.042 | -0.001 (-0.004, 0.002) | 0.486 |

* N=3 patients

Table S19 multivariable adjusted R-squared: 0.5882

# Table S20. Factors associated with time to haemoglobin recovery, including primaquine *C*_max_ (N=258).

| **Variables** | **Univariate analysis** | | **Multivariable analysis** | |
| --- | --- | --- | --- | --- |
|  | **HR (95% CI)** | **p-value** | **Adjusted HR (95% CI)** | **p-value** |
| Age (years) | 0.98 (0.94, 1.01) | 0.297 | 1.05 (1.00, 1.10) | 0.041 |
| Sex  Male (reference)  Female | 1.02 (0.78, 1.33) | 0.880 | NA | NA |
| Length of illness before treatment (days) | 1.05 (0.96, 1.15) | 0.261 | NA | NA |
| Mid-upper arm circumference (cm) | 0.96 (0.90, 1.02) | 0.176 | NA | NA |
| Fractional fall (%) in haemoglobin | 0.93 (0.86, 1.00) | 0.072 | NA | NA |
| Baseline haemoglobin (g/dL) | 0.77 (0.71, 0.83) | <0.001 | 0.73 (0.66, 0.79) | <0.001 |
| Splenomegaly  Normal (reference) | 1.35 (0.99, 1.84) | 0.059 | NA | NA |
| Hepatomegaly  Normal (reference) | 1.48 (0.76, 2.89) | 0.248 | NA | NA |
| Sickle cell status  Normal (HbAA) (reference) |  |  |  |  |
| Trait (HbAS) | 1.21 (0.79, 1.84) | 0.384 | NA | NA |
| Disease (HbSS) * | 0.59 (0.15, 2.37) | 0.457 | NA | NA |
| Thalassemia status  Normal (αα/αα) (reference) |  |  |  |  |
| Heterozygous (-α/αα) | 1.01 (0.77, 1.33) | 0.907 | 0.88 (0.65, 1.20) | 0.425 |
| Homozygous (-α/-α) | 1.72 (1.09, 2.73) | 0.019 | 1.11 (0.67, 1.86) | 0.685 |
| G6PD  Normal (reference) |  |  |  |  |
| Heterozygous females | 0.85 (0.52, 1.37) | 0.503 | NA | NA |
| Deficient males and females | 1.15 (0.52, 1.58) | 0.376 | NA | NA |
| Log of baseline parasitaemia | 0.90 (0.86, 0.95) | <0.001 | 0.89 (0.84, 0.93) | <0.001 |
| Treatment failure  ACPR (reference) | 1.04 (0.64, 1.68) | 0.880 | NA | NA |
| *C*max (ng/mL) | 1.00 (1.00, 1.00) | 0.618 | NA | NA |

* N=3 patients

Table S20 Akaike Information Criterion (AIC): 1703.44

# Table S21 (A) Mean /Median /IQR /Range for haemoglobin parameters (Total N=1,137).

| **Variable** | **N** | **SD** | **Mean** | **Min** | **p25** | **p50** | **p75** | **Max** |
| --- | --- | --- | --- | --- | --- | --- | --- | --- |
| **Time to HB recovery** | 1,137 | 14.3 | 15.3 | 0.3 | 2.0 | 14.0 | 21.0 | 42.0 |
| *By G6PD status* |  |  |  |  |  |  |  |  |
| G6PD Normal | 717 | 15.0 | 16.2 | 0.3 | 2.0 | 14.0 | 28.0 | 42.0 |
| G6PD heterozygous females | 119 | 14.1 | 16.3 | 0.3 | 2.5 | 14.0 | 28.0 | 42.0 |
| G6PDd males and females | 284 | 12.0 | 12.5 | 0.3 | 2.0 | 7.0 | 21.0 | 42.0 |
| Total | 1,120 | 14.3 | 15.2 | 0.3 | 2.0 | 14.0 | 21.0 | 42.0 |
|  |  |  |  |  |  |  |  |  |
| **Nadir haemoglobin** | 1,137 | 1.5 | 9.2 | 4.6 | 8.2 | 9.4 | 10.3 | 13.2 |
| *By G6PD status* |  |  |  |  |  |  |  |  |
| G6PD Normal | 717 | 1.5 | 9.3 | 4.6 | 8.4 | 9.5 | 10.4 | 13.2 |
| G6PD heterozygous females | 119 | 1.5 | 9.1 | 5.7 | 8.0 | 9.3 | 10.2 | 11.8 |
| G6PDd males and females | 284 | 1.5 | 9.1 | 4.8 | 8.0 | 9.1 | 10.1 | 12.4 |
| Total | 1,120 | 1.5 | 9.2 | 4.6 | 8.2 | 9.4 | 10.3 | 13.2 |
|  |  |  |  |  |  |  |  |  |
| **Fractional Fall (%) in haemoglobin** | 1,137 | 8.6 | 12.8 | 0 | 6.8 | 11.7 | 17.5 | 57.9 |
| *By G6PD status* |  |  |  |  |  |  |  |  |
| G6PD Normal | 717 | 8.6 | 12.9 | 0 | 7.0 | 11.9 | 17.7 | 57.9 |
| G6PD heterozygous females | 119 | 8.3 | 13.1 | 0 | 7.1 | 13.3 | 17.8 | 37.0 |
| G6PDd males and females | 284 | 8.3 | 11.8 | 0 | 5.7 | 10.0 | 16.6 | 40.4 |
| Total | 1,120 | 8.5 | 12.7 | 0 | 6.8 | 11.7 | 17.5 | 57.9 |
|  |  |  |  |  |  |  |  |  |
| **Haemoglobin concentration on D42** | 1,066 | 1.1 | 11.7 | 6.1 | 11.1 | 11.9 | 12.5 | 16.4 |
| *By G6PD status* |  |  |  |  |  |  |  |  |
| G6PD Normal | 668 | 1.2 | 11.7 | 6.1 | 11.0 | 11.9 | 12.5 | 16.4 |
| G6PD heterozygous females | 113 | 1.2 | 11.7 | 7.3 | 10.9 | 11.8 | 12.5 | 13.7 |
| G6PDd males and females | 270 | 1.0 | 11.9 | 7.9 | 11.3 | 12.0 | 12.5 | 14.1 |
| Total | 1,051 | 1.1 | 11.7 | 6.1 | 11.1 | 11.9 | 12.5 | 16.4 |
|  |  |  |  |  |  |  |  |  |
| **Malaria attributable fraction** | 1,066 | 1.4 | 2.5 | 0 | 1.5 | 2.4 | 3.4 | 8.0 |
| *By G6PD status* |  |  |  |  |  |  |  |  |
| G6PD Normal | 668 | 1.4 | 2.4 | 2.2 | 1.4 | 3.2 | 0 | 8.0 |
| G6PD heterozygous females | 113 | 1.5 | 2.7 | 0 | 1.7 | 2.4 | 3.6 | 6.0 |
| G6PDd males and females | 270 | 1.4 | 2.8 | 0.3 | 1.8 | 2.6 | 3.7 | 7.7 |
| Total | 1,051 | 1.4 | 2.5 | 0 | 1.5 | 2.4 | 3.4 | 8.0 |
|  |  |  |  |  |  |  |  |  |
